# Altered longevity-assurance activity of p53:p44 in the mouse causes memory loss, neurodegeneration and premature death

**DOI:** 10.1111/j.1474-9726.2010.00547.x

**Published:** 2010-04

**Authors:** Mariana Pehar, Kenneth J O’Riordan, Melissa Burns-Cusato, Matthew E Andrzejewski, Carlos Gil del Alcazar, Corinna Burger, Heidi Scrable, Luigi Puglielli

**Affiliations:** 1 Department of Medicine, University of Wisconsin-Madison2500 Overlook Terrace, Madison, WI 53705, USA; 2 Department of Neurology, University of Wisconsin-Madison1215 Linden Dr, Madison, WI 53706, USA; 3 Department of Neuroscience, University of VirginiaBox 801392, Charlottesville, VA 22908, USA; 4 Rodent Models Core, Waisman Center, 1500 Highland Ave, University of Wisconsin-MadisonWI 53705, USA; 5 Geriatric Research Education Clinical Center, VA Medical Center2500 Overlook Terrace, Madison, WI 53705, USA

**Keywords:** Alzheimer’s disease, insulin-like growth factor 1 receptor, memory loss, neurodegeneration, p44, p53

## Abstract

The longevity-assurance activity of the tumor suppressor p53 depends on the levels of Δ40p53 (p44), a short and naturally occurring isoform of the *p53* gene. As such, increased dosage of p44 in the mouse leads to accelerated aging and short lifespan. Here we show that mice homozygous for a transgene encoding p44 (p44^+/+^) display cognitive decline and synaptic impairment early in life. The synaptic deficits are attributed to hyperactivation of insulin-like growth factor 1 receptor (IGF-1R) signaling and altered metabolism of the microtubule-binding protein tau. In fact, they were rescued by either *Igf1r* or *Mapt* haploinsufficiency. When expressing a human or a ‘humanized’ form of the amyloid precursor protein (APP), p44^+/+^ animals developed a selective degeneration of memory-forming and -retrieving areas of the brain, and died prematurely. Mechanistically, the neurodegeneration was caused by both paraptosis- and autophagy-like cell deaths. These results indicate that altered longevity-assurance activity of p53:p44 causes memory loss and neurodegeneration by affecting IGF-1R signaling. Importantly, *Igf1r* haploinsufficiency was also able to correct the synaptic deficits of APP_695/swe_ mice, a model of Alzheimer’s disease.

## Introduction

Studies from model organisms have shown that an aging program mediated by the insulin-like growth factor 1 receptor (IGF-1R) regulates lifespan. In fact, hyperactivation of IGF-1R signaling leads to accelerated aging and short lifespan, whereas partial inhibition of IGF-1R delays aging and extends lifespan (Tissenbaum & Guarente, 2002; Kenyon, 2005; Puglielli, 2008; Ungewitter & Scrable, 2008). Additionally, genetic variations that cause reduced IGF-1R signaling appear to be beneficial for old age survival in humans (van Heemst *et al.*, 2005; Suh *et al.*, 2008), suggesting that the mechanisms regulating lifespan and aging *via* this pathway are highly conserved.

In mammals, IGF-1R signaling is tightly controlled by the longevity-assurance activity of the tumor suppressor p53, specifically Δ40p53 (Ungewitter & Scrable, 2008). Δ40p53 (also referred to as ΔNp53 or p44) is one of several N-truncated isoforms encoded by the *p53* gene, which lack the first transactivation domain (Courtois *et al.*, 2002; Yin *et al.*, 2002; Ghosh *et al.*, 2004; Bourdon *et al.*, 2005). It has become evident that the biological functions of wild-type p53 do not simply depend on the expression levels of the full-length protein but also on the interplay between the different isoforms (Courtois *et al.*, 2002; Yin *et al.*, 2002; Ghosh *et al.*, 2004; Bourdon *et al.*, 2005).

In genetically altered mice, truncation of the N-terminus of full-length p53 (p53^+/m^ mice) (Tyner *et al.*, 2002) or increased dosage of Δ40p53 (p44^+/+^ mice) (Maier *et al.*, 2004) results in premature and accelerated aging and in reduced longevity. In p44^+/+^ mice, this has been linked to altered function of full-length p53 and to the consequent hyperactivation of IGF-1R signaling. In fact, a delicate balance between p53 and Δ40p53 (p44) is thought to control both expression levels and signaling activity of IGF-1R (Bauer & Helfand, 2006; Rodier *et al.*, 2007; Ungewitter & Scrable, 2008). Increased activity of Δ40p53 (p44) in the mouse tilts the balance toward hyperactivation of IGF-1R signaling and leads to accelerated aging (Maier *et al.*, 2004). Importantly, the deletion of p53 in p44^+/+^ mice abolishes the progeroid phenotype of the animals (Maier *et al.*, 2004), indicating that it is the p53:p44 system and not Δ40p53 (p44) alone that regulates the aging program. To date, a total of six mouse models with altered p53 activity have been generated, and in all of them age-associated events and lifespan are altered. Collectively, they leave little doubt that the p53:p44 system has longevity-assurance activity (Bauer & Helfand, 2006; Rodier *et al.*, 2007; Ungewitter & Scrable, 2008).

Here, we report that altered longevity-assurance activity of the p53:p44 system in p44^+/+^ transgenic mice leads to cognitive decline and synaptic impairment. The synaptic deficits are attributed to hyperactivation of IGF-1R signaling and altered metabolism of the microtubule-binding protein tau, and can be rescued by *Igf1r* or *Mapt* haploinsufficiency. Additionally, when crossed with mice expressing a ‘humanized’ form of mouse amyloid precursor protein (APP), p44^+/+^ animals develop a selective degeneration of memory-forming and -retrieving areas of the brain and die prematurely. The neuronal degeneration is caspase- and apoptosis-independent and involves co-activation of paraptosis- and autophagy-like cell death. Finally, we show that haploinsufficiency of *Igf1r* is also able to correct the synaptic deficits of APP_695/swe_ mice, a commonly used model of Alzheimer’s disease (AD).

## Results

### p44^+/+^ mice show memory impairment and synaptic defects as a result of IGF-1R hyperactivation and abnormal tau metabolism

**Fig. 1:**
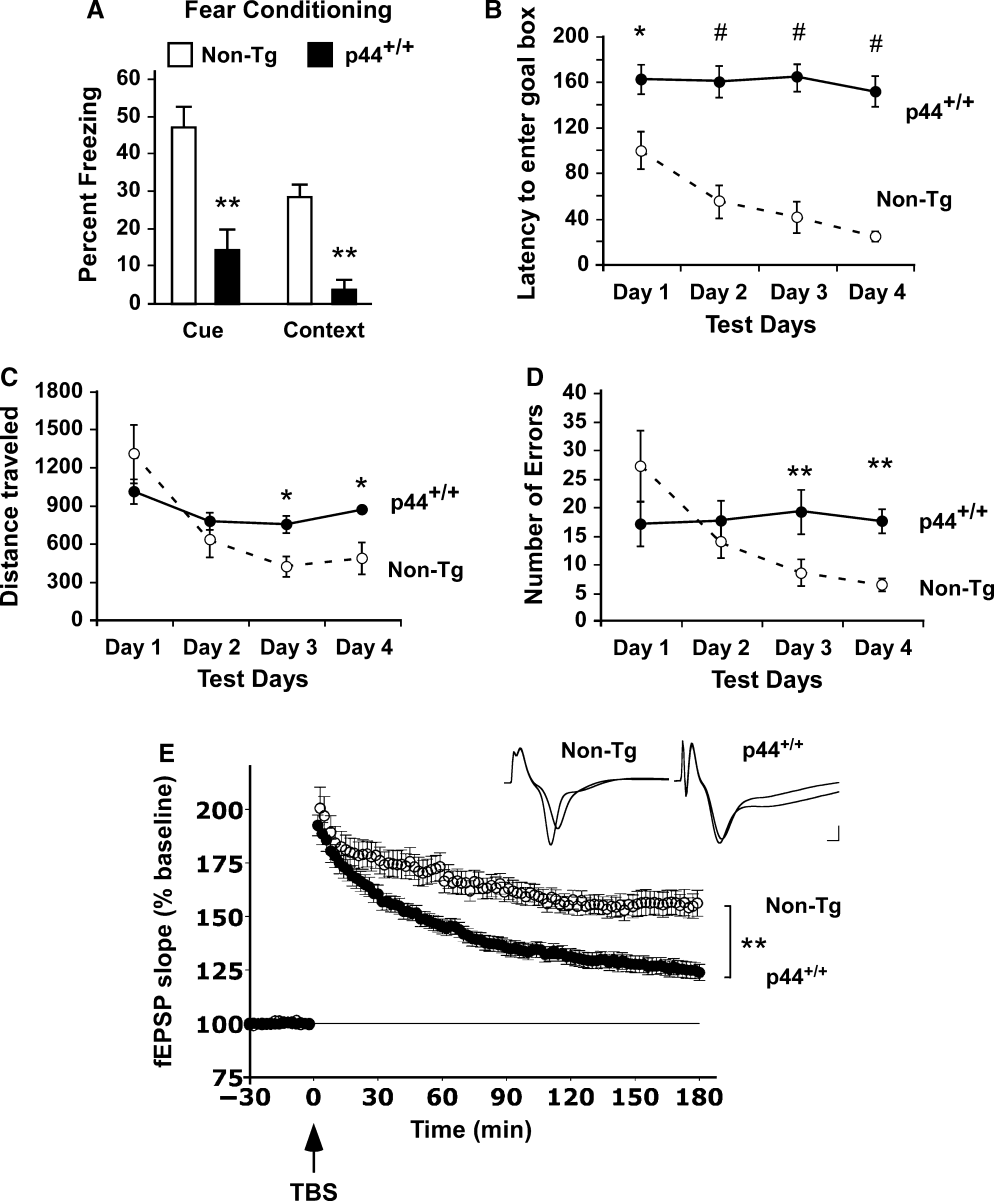
p44^+/+^ single-transgenics display memory and synaptic defects. (A) Fear conditioning assessment of 2.5-month-old male p44^+/+^ (*n*= 9) and nontransgenic (non-Tg; *n*= 14) littermates. Results show percentage of freezing. (B–D) Barnes maze assessment of 10-month-old male p44^+/+^ and non-Tg littermates showing latency to enter in the goal box (B, *n*= 12, both groups), distance traveled (C, *n*= 6, both groups) and number of errors (D, *n*= 12, both groups). (E) Long-term potentiation (LTP) induction in hippocampal slices of 2.5-month-old male p44^+/+^ and non-Tg littermates. p44^+/+^ [*n*= 37(12)] lack the late component of LTP seen in non-Tg mice [*n*= 11(5)]. *Inset*: typical recordings at the beginning (prior to stimulation) and end of the experiments. Calibration: 1 mV, 1 ms. The basal synaptic excitatory transmission, as assessed by paired-pulse facilitation (PPF) and i/o curves, is shown in Fig. S2. All values are mean ± SEM. *P < 0.05, **P < 0.005, #*P* < 0.0005.

To assess whether overexpression of p44 in the mouse causes cognitive decline, we performed a systematic evaluation of p44^+/+^ transgenic mice by using a combination of cognitive, electrophysiological, and histological approaches. As shown in Fig. 1A, p44^+/+^ mice displayed a marked defect in associative memory retrieval, as assessed by contextual- and cued-fear conditioning tests, which explore amygdala and hippocampal functions. The memory impairment occurred very early in life (already evident at the age of 2.5 months) and in the absence of general locomotor or behavioral abnormalities, as assessed with the open-field test (Fig. S1). Similar deficits were observed in older mice with the Barnes maze test. The Barnes maze is more specific for spatial navigational memory, which is a hippocampal-dependent activity. p44^+/+^ transgenic animals displayed increased latency in entering the goal box (Fig. 1B), increased distance traveled (Fig. 1C), and increased number of errors (Fig. 1D). Importantly, p44^+/+^ mice did not show any significant improvement on the spatial navigational task with training (Fig. 1B–D). In conclusion, the cognitive defects of p44^+/+^ mice were observed on different experimental paradigms and were evident throughout their entire lives.

It has been suggested that negative changes in the postsynaptic component of the long-term potentiation (LTP), as assessed in hippocampal brain slices, represent the electrophysiological correlate of the learning decline that characterizes aging in animals (Hedden & Gabrieli, 2005; Yankner *et al.*, 2008). Therefore, we measured CA3-CA1 LTP, as induced by theta burst stimulation (TBS) to the Schaffer collaterals, in hippocampal slices from 2.5-month-old p44^+/+^ and nontransgenic (non-Tg) littermates. p44^+/+^ transgenics exhibited normal induction of the field excitatory postsynaptic potential (fEPSP) slope (Fig. 1E). However, after the initial 30 min, this transient potentiation decayed drastically (relative to non-Tg littermates), indicating that p44^+/+^ mice lack the late component of LTP. The paired-pulse facilitation (PPF), a measure of presynaptic contribution to synaptic transmission and a known form of short-term synaptic plasticity, did not differ from non-Tg littermates (Fig. S2), indicating that p44^+/+^ mice only lack the postsynaptic component of LTP. The electrophysiological deficits of the hippocampal brain slices (Fig. 1E) reflect very well the cognitive defects of the living animals (Fig. 1A-D).

**Fig. 2:**
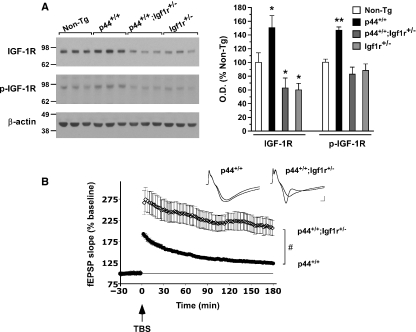
IGF-1R is responsible for the synaptic deficits of p44^+/+^ animals. (A) Expression levels of IGF-1R and phospho-IGF-1R (p-IGF-1R) in the hippocampal formation of 2.5-month-old male non-Tg, p44^+/+^, p44^+/+^;Igf1r^+/−^, and Igf1r^+/−^ animals. A representative Western blot of three different animals is shown in the left panel, whereas the percentage of change is shown in the right panel (*n*= 4). (B) Enhanced hippocampal CA1 long-term potentiation (LTP) in 2.5-month-old male p44^+/+^;Igf1r^+/−^ transgenic animals. Theta burst stimulation (TBS)-induced LTP was enhanced in p44^+/+^;Igf1r^+/−^ mice [*n*= 10(3)] when compared to p44^+/+^ single-transgenics [*n*= 37(12)]. *Insets*: example traces before and after LTP for each group. Calibration 1 mV, 1 ms. No difference was observed in paired-pulse facilitation (PPF) or i/o curves (data not shown). All values are mean ± SEM. *P < 0.05; **P < 0.005, #*P* < 0.0005.

Dysregulation of the longevity-assurance activity of the p53:p44 system, as caused by the overexpression of Δ40p53 (p44), leads to hyperactivation of IGF-1R signaling (Maier *et al.*, 2004), which in turn is thought to cause the progeroid phenotype of p44^+/+^ animals (Bauer & Helfand, 2006; Rodier *et al.*, 2007; Ungewitter & Scrable, 2008). Consistently, p44^+/+^ transgenic mice displayed increased levels of both IGF-1R and phospho-IGF-1R, the activated form of the receptor, in the hippocampal formation (Fig. 2A). To assess whether the synaptic deficits displayed by p44^+/+^ animals were directly related to the ability of Δ40p53 (p44) to activate IGF-1R signaling, we crossed p44^+/+^ single-transgenics with mice lacking one copy of *Igf1r* (Igf1r^+/−^) and generated p44^+/+^;Igf1r^+/−^ double-transgenics. Disruption of both copies of *Igf1r* (Igf1r^−/−^) is embryonically lethal whereas disruption of only one copy (Igf1r^+/−^) extends lifespan in the absence of obvious pathological manifestations (Holzenberger *et al.*, 2003). Assessment of the synaptic transmission and plasticity properties of p44^+/+^;Igf1r^+/−^ double-transgenics revealed a significant increase in both induction and maintenance of the LTP of the field excitatory postsynaptic potential slope when compared to p44^+/+^ single-transgenics, thus indicating that the deficits observed in p44^+/+^ animals were caused by hyperactivation of IGF-1R signaling (Fig. 2B; also discussed later).

**Fig. 3:**
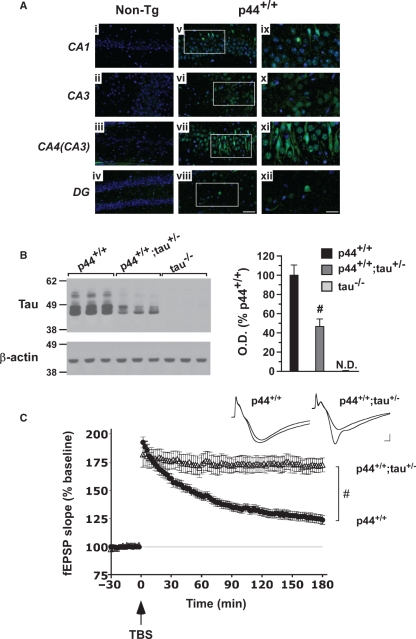
The abnormal metabolism of tau is responsible for the synaptic deficits of p44^+/+^ animals. (A) Immunostaining with AT8 antibody shows abnormal tau phosphorylation (Ser202 and Thr205) in the indicated subfields of the hippocampus and dentate gyrus of p44^+/+^ mice (*v-xii*). No staining is observed in non-Tg mice (*i-iv*). (*ix-xii*) show higher magnification of the indicated areas in (*v-viii*). AT8 immunoreactivity was mainly localized in neuronal cell bodies. Nuclei were counterstained with 4′,6-diamidino-2-phenylindole (DAPI) (blue). Scale bars: 40 μm (*i-viii*); 20 μm (*ix-xii*). Animals were 2.5-month old when analyzed. Immunostaining with a different anti-phospho-tau antibody is shown in Fig. S7. (B) Expression levels of tau in the hippocampal formation of 2.5-month-old male p44^+/+^ and p44^+/+^;tau^+/−^ animals. A representative Western blot of three different animals is shown in the left panel, whereas the percentage of change is shown in the right panel (*n*= 4). Tau^−/−^ mice are included as control for antibody specificity. ND: not detected. (C) Enhanced hippocampal CA1 long-term potentiation (LTP) in 2.5-month-old male p44^+/+^;tau^+/−^ transgenic animals. Theta burst stimulation (TBS)-induced LTP was enhanced in p44^+/+^;tau^+/−^ mice [*n*= 7(4)] when compared to p44^+/+^ single-transgenics [*n*= 37(12)]. *Insets*: example traces before and after LTP for each transgenic group. Calibration 1 mV, 1 ms. No difference was observed in paired-pulse facilitation (PPF) or i/o curves (data not shown). All values are mean ± SEM. #*P* < 0.0005.

Histological assessment of different regions of the central nervous system of p44^+/+^ transgenic animals revealed no abnormalities (see later), with the exception of the hyperphosphorylation of the microtubule-binding protein tau (Fig. 3A; see also Fig. S7B). The anti-phospho-tau immunolabeling was performed with different antibodies recognizing different ‘phospho-epitopes’ (p-Ser202, p-Thr205 and p-Ser356; see Figs 3A and S7B). Importantly, phospho-tau immunoreactivity exhibited a somatodendritic distribution, thus resembling the typical ‘pretangle’ lesion observed in the brain of nondemented old individuals and of patients affected by late-onset AD (Bancher *et al.*, 1989). It is worth noting that mouse tau differs from human both in the primary sequence and in the alternative splicing of the different isoforms. As a consequence, mouse tau does not generate the classical paired helical filaments or neurofibrillary tangles that are observed in the brain of nondemented old individuals and of patients affected by late-onset AD (Duyckaerts *et al.*, 2008).

Both AD and non-AD mouse models indicate that the abnormal metabolism of tau can cause memory deficits [reviewed in (Lee *et al.*, 2001)]. Additionally, mutations in the gene encoding tau (*Mapt*) have been associated with frontotemporal forms of dementia [reviewed in (Lee *et al.*, 2001)]. Therefore, we analyzed whether tau was responsible for the synaptic deficits observed in p44^+/+^ single-transgenics by studying the synaptic transmission and plasticity properties of p44^+/+^;tau^+/−^ animals.

As expected, p44^+/+^;tau^+/−^ double-transgenic mice displayed a ∼50% decrease in tau protein levels (Fig. 3B). LTP assessment revealed that haploinsufficiency of the *Mapt* gene in p44^+/+^;tau^+/−^ double-transgenics was able to correct the deficiency in the late component of LTP observed in p44^+/+^ single-transgenics (Fig. 3C). It should be noted that *Mapt* haploinsufficiency only improved the late component of LTP whereas *Igf1r* hypoinsufficiency enhanced both the induction phase and the late component of LTP (compare Figs 3C and 2B). The early and late components of LTP are thought to differ in the fact that the former is protein and RNA synthesis-independent whereas the latter is protein and RNA synthesis-dependent. Therefore, it is likely that IGF-1R signaling affects multiple aspects of synaptic transmission, some of which are tau-independent. It is also possible that compensatory mechanisms are responsible for the apparent increase in the induction phase in p44^+/+^;Igf1r^+/−^ double-transgenics (see also later).

### Expression of a humanized form of mouse APP in p44^+/+^ mice causes reduced lifespan and massive degeneration of memory-forming and -retrieving areas of the brain

**Fig. 4:**
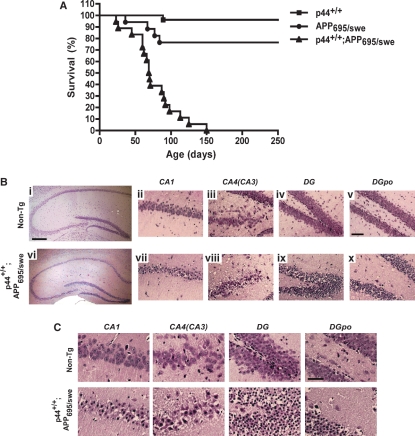
Decreased lifespan and widespread degeneration of the hippocampal formation in double-transgenic p44^+/+^;APP_695/swe_ mice. (A) Kaplan–Meier survival analysis for p44^+/+^ (squares), APP_695/swe_ (circles) and double-transgenic p44^+/+^;APP_695/swe_ (triangles) mice. The median survival time for p44^+/+^;APP_695/swe_ mice was 69.5 days. In the double-transgenic group (*n*= 19) no animal survived longer than 5 months. At this time, the survival in the APP_695/swe_ (*n*= 16) and p44^+/+^ (*n*= 26) groups was 76.5% and 96.1%, respectively. The survival curves were significantly different (*P* < 0.0005). (B) Hematoxylin and eosin stained brain sections of 2.5-month-old male non-Tg and p44^+/+^;APP_695/swe_ double-transgenic mice. Bar in left panel (2.5×): 400 μm; bar in right panel (10×): 100 μm. (C) Higher-magnification images of (B). Bar: 50 μm. APP_695/swe_ and p44^+/+^ animals are shown in Fig. S5. *DG*: Dentate Gyrus; *DGpo*: polymorph layer of the dentate gyrus; *CA4(CA3)* indicates the part of *CA3* that inserts into the dentate gyrus.

To investigate whether IGF-1R hyperactivation, as caused by altered longevity-assurance activity of p53:p44, could cause AD-like neurodegeneration *in vivo*, we bred homozygous p44-transgenic mice (p44^+/+^) with heterozygous APP_695/swe_ mice, which express a modified mouse APP cDNA encoding the 695-amino acid isoform with a ‘humanized’ amyloid β-peptide (Aβ) domain that includes the familial AD-associated Swedish double mutation (K595N/M596L). APP_695/swe_ mice develop amyloid plaques in advanced age but lack significant memory loss or neurodegeneration (Borchelt *et al.*, 1996; Savonenko *et al.*, 2003). The resulting p44^+/+^;APP_695/swe_ double-transgenic animals were born with mendelian frequency and without visible defects. Remarkably, they displayed a very short lifespan, with a median survival of 2.3 months (Fig. 4A). At the moment of death, the peripheral organs appeared normal with no obvious signs of pathology/degeneration (Fig. S3). The only abnormalities that we could identify were limited to the brain (see later).

**Fig. 5:**
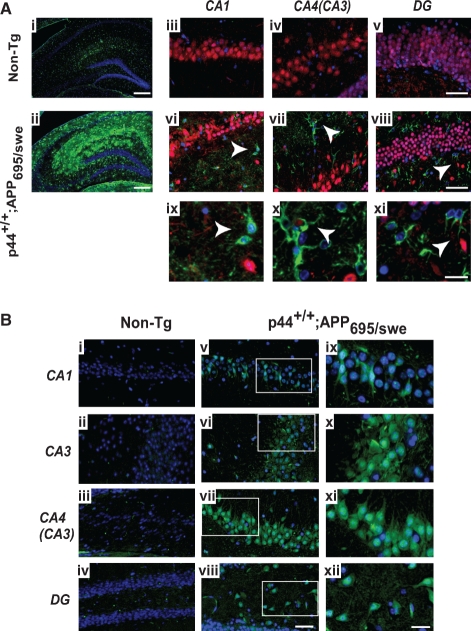
Widespread astrogliosis and tau hyperphosphorylation in the hippocampus and dentate gyrus of p44^+/+^;APP_695/swe_ double-transgenic mice. (A) Brain sections of 2.5-month-old non-Tg and p44^+/+^;APP_695/swe_ double-transgenic mice were immunostained for GFAP (astrocytic marker; green) and NeuN (neuronal marker; red). Nuclei were counterstained with 4′,6-diamidino-2-phenylindole (DAPI) (blue). (*i* and *ii*) Low-magnification microphotographs showing GFAP immunoreactivity in the hippocampus and dentate gyrus of non-Tg and double-transgenic animals. (*iii-viii*) Higher magnification of CA1, CA4 and dentate gyrus (DG) of non-Tg (*iii-v*) and double-transgenic (*vi-viii*) mice. Arrowheads indicate the characteristic morphology of reactive astrocytes with hypertrophic soma and thick processes surrounding degenerating neurons. (*ix-xi*) Higher magnification of reactive astrocytes indicated in (*vi-viii*). Scale bars: 400 μm (*i* and *ii*); 50 μm (*iii-viii*) and 20 μm (*ix-xi*). APP_695/swe_ and p44^+/+^ animals are shown in Fig. S6. (B) Immunostaining with AT8 antibody showing abnormal tau phosphorylation (Ser202 and Thr205) in the indicated subfields of the hippocampus and dentate gyrus from double-transgenic mice (*v-viii*). (*ix-xii*) Higher magnification of the indicated areas in (*v-viii*). AT8 immunoreactivity was mainly localized in neuronal cell bodies. Nuclei were counterstained with DAPI (blue). Images (*i-iv*) are also shown in Fig. 3A. Scale bars: 40 μm (*i-viii*); 20 μm (*ix-xii*). APP_695/swe_ and p44^+/+^ animals, as well as immunostaining with a different anti-phospho-tau antibody, are shown in Fig. S7. Animals were 2.5-month old when analyzed.

**Fig. 6:**
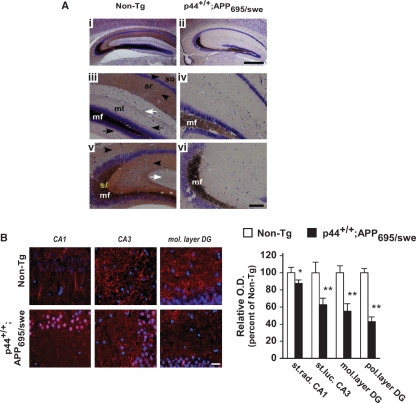
Early loss of synaptic terminals in p44^+/+^;APP_695/swe_ double-transgenic mice. (A) Timm’s sulfide silver staining of coronal brain sections of 1.5-month-old non-Tg (*i*, *iii*, *v*) and p44^+/+^;APP_695/swe_ mice (*ii*, *iv*, *vi*). Images (*iii*) to (*vi*) show higher magnification of (*i*) and (*ii*). When compared to non-Tg, double-transgenic animals displayed a severe loss of Timm’s staining. Specifically, the mossy fibers (mf) showed a reduced staining whereas the ipsilateral and commissural hippocampodentate pathways (black arrows), the commissural and ipsilateral association systems in the CA1-CA3 *stratum oriens* and *stratum radiatum* (black arrow-heads), and the lateral perforant path (white arrows) could not be stained at all. Abbreviations: mf, mossy fibers; ml, dentate molecular layer; *sl*, CA3 *stratum lucidum*; *so*, *stratum oriens*; *sr*, *stratum radiatum*. Scale bars: 500 μm (*i* and *ii*) and 100 μm (*iii-vi*). APP_695/swe_ and p44^+/+^ animals are shown in Fig. S8. (B) Representative microphotographs showing synaptophysin immunoreactivity (red) in brain sections of non-Tg and p44^+/+^;APP_695/swe_ mice. Nuclei were counterstained with 4′,6-diamidino-2-phenylindole (DAPI) (blue). The ‘synaptic boutons’ are well visible in non-Tg mice but significantly reduced in double-transgenic mice. Scale bar: 25 μm. Graphic in right panel shows relative optical density (OD) quantification of synaptophysin immunoreactivity from CA1 stratum radiatum (st. rad.), CA3 stratum lucidum (st. luc.), molecular layer of dentate gyrus (mol. layer DG) and polymorph layer of dentate gyrus (pol. layer DG). Values are mean ± SEM. *P < 0.05, **P < 0.005.

**Fig. 9:**
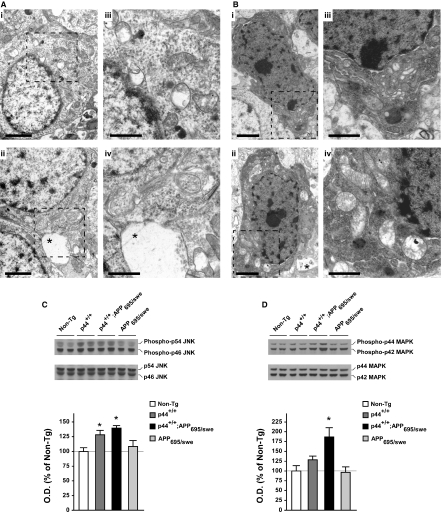
Both paraptosis- and autophagy-like events are responsible for the neuronal degeneration in p44^+/+^;APP_695/swe_ double transgenics. (A) Electron micrographs showing neurons undergoing paraptosis-like degeneration (*i* and *ii*). These neurons appeared lightly stained and preserved their overall morphology with normal nuclei and no evidence of chromatin condensation. Their cytoplasm displayed vacuoles and swollen mitochondria. *iii* and *iv* show higher magnification of the indicated areas in *i* and *ii*, respectively. p44^+/+^;APP_695/swe_ double-transgenic mice also displayed dystrophic processes surrounding degenerating neurons (*, asterisk). Dilated ER (*iv*) and mitochondria (*iii* and *iv*), together with several cytoplasmic inclusions (*iii* and *iv*), are evident. The swollen mitochondria showed ruptured inner membranes (*iii* and *iv*). Scale bar: 2 μm for *i* and *ii*; 1 μm for *iii* and *iv*. Low-magnification images are shown in Fig. S9. Animals were 2.5-month old when analyzed. (B) Electron micrographs showing neurons undergoing dark cell degeneration (*i* and *ii*). They were strongly stained with osmium and displayed irregular morphology with highly electron dense cytoplasm containing dense vacuoles, swollen mitochondria and dilated ER-Golgi network. The mitochondria had very few cristae and exhibited rupture of the inner membrane. The nucleus appeared highly condensed with crenated nucleolemma and chromatin clustered in peripheral bundles. *iii* and *iv* show higher magnification of the indicated areas in *i* and *ii*, respectively. Scale bar: 2 μm for *i* and *ii*; 1 μm for *iii* and *iv*. Low-magnification images are shown in Fig. S9. Animals were 2.5-month old when analyzed. (C and D) Jun N-terminal kinases (JNK) (C) and mitogen-activated protein kinases (MAPK) (D) phosphorylation in the hippocampus of non-Tg, p44^+/+^, APP_695/swe_ and p44^+/+^;APP_695/swe_ mice. A representative Western blot of two different animals is shown in the upper panel whereas the percentage of change is shown in the lower panel (*n*= 4). The levels of JNK and MAPK phosphorylation were normalized against the levels of total JNK or MAPK protein, respectively. Animals were 2.5-month old when analyzed. Results are expressed as percentage of non-Tg and are the mean ± SEM. *P < 0.05.

The external layers of the neocortex in p44^+/+^;APP_695/swe_ mice showed sparse eosinophilic neurons with dense cytoplasm (Fig. S4A) and surrounded by few reactive astrocytes (Fig. S4B). The cerebellum and the spinal cord appeared completely normal and similar to non-Tg animals (Fig. S4A). The analysis of the hippocampus and dentate gyrus (*fascia dentata*) revealed normal cyto-architecture (see low-magnification images in the left panel of Fig. 4B) excluding possible defects in migration and/or differentiation of the neurons during development. This is consistent with the fact that the double-transgenic animals did not display signs of neurodegeneration at birth (data not shown). However, the entire hippocampal formation of p44^+/+^;APP_695/swe_ mice suffered from massive and widespread degeneration. In fact, dense, shrunken, and highly eosinophilic (darkly stained) neurons were extremely abundant and well evident throughout the entire hippocampus and dentate gyrus (Fig. 4B,C; for p44^+/+^ and APP_695/swe_ single-transgenics see Fig. S5). They co-existed with large vacuoles, thus resembling a ‘vacuolar-like’ form of neurodegeneration (see also later in Fig. 9). Importantly, the above phenotype was only evident in the double-transgenics as the other littermates, including p44^+/+^ and APP_695/swe_ single-transgenics appeared normal and similar to non-Tg mice (Fig. S5). The immunoreactivity to the astrocytic marker GFAP revealed widespread astrogliosis (Fig. 5A), which appeared much more severe in the areas that correspond to the synaptic fields of the lateral perforant pathway and the commissural and ipsilateral association systems (see also later in Figs 6 and S8). This finding is consistent with the widespread degeneration shown in Fig. 4 and was absent in age-matched non-Tg, p44^+/+^, and APP_695/swe_ littermates (Fig. S6). Finally, the hippocampus and dentate gyrus of p44^+/+^;APP_695/swe_ mice also displayed abnormal phosphorylation of the microtubule-associated protein tau (Figs 5B and S7). Interestingly, the distribution pattern of tau hyperphosphorylation in p44^+/+^;APP_695/swe_ double-transgenics was similar to that observed in p44^+/+^ single-transgenics (Fig. S7).

To analyze early changes in the synaptic organization of the hippocampus and dentate gyrus, we performed Timm’s sulfide silver staining, which allows the identification of the abundant zinc-containing synaptic terminals. Non-Tg, APP_695/swe_ and p44^+/+^ single-transgenic animals displayed the characteristic laminar Timm’s staining (Figs 6A and S8), which directly correlates with the synaptic fields of the different afferent systems (for a detailed description see Fig. S8). In contrast, p44^+/+^;APP_695/swe_ mice showed a severe loss of staining in the areas corresponding to the commissural/ipsilateral association systems and to the perforant pathway, which conduct information from the entorhinal cortex to the dentate gyrus and CA3 areas of the hippocampus (Fig. 6A; for p44^+/+^ and APP_695/swe_ single-transgenics see Fig. S8). Only the mossy fibers, which project from the granule cells of the dentate gyrus to CA3 *via* the polymorphic layer (*hilus*), could be visualized in the double-transgenic animals, although less intensely than in non-Tg littermates (Fig. 6A). Neither p44^+/+^ nor APP_695/swe_ mice displayed any alteration in the intensity or pattern of staining (Fig. S8). The loss of Timm’s staining was already evident at 1.5 months of age and represented the earliest alteration that we could detect, before widespread degeneration of the hippocampus and dentate gyrus. These results suggest a functional disconnection of the different subregions of the hippocampal formation, which has also been described as a typical neuropathological feature and a possible cause of the early memory loss in patients with AD (Hyman *et al.*, 1984).

The loss of Timm’s staining can indicate either functional alterations or nonreversible degeneration of the synaptic terminals. To distinguish between these possibilities, we immunolabeled the same hippocampal areas with an antibody against the integral synaptic vesicle protein, synaptophysin. Figure 6(B) shows an abundant and healthy synaptic network in non-Tg animals, which was significantly reduced in p44^+/+^;APP_695/swe_ mice. These alterations are consistent with the phenotype described earlier and support the conclusion that the loss of Timm’s staining is because of the degeneration of the synaptic terminals.

**Fig. 7:**
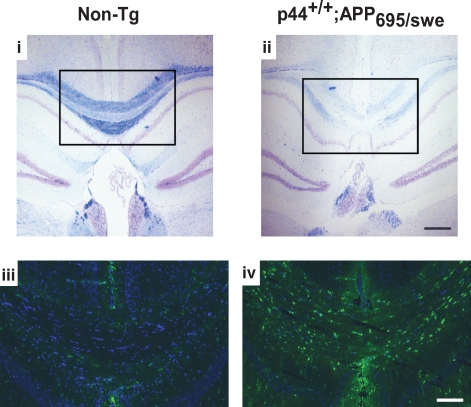
Degeneration of the corpus callosum in p44^+/+^;APP_695/swe_ double-transgenic mice. Luxol fast blue-Cresyl violet (Klüver–Barrera) staining of 2.5-month-old non-Tg and p44^+/+^;APP_695/swe_ mice. Coronal brain sections at comparable anteroposterior levels are shown. Immunostaining for GFAP (green; *iii*, *iv*) of areas indicated in (*i*) and (*ii*) reveals that the myelin loss in p44^+/+^;APP_695/swe_ mice is accompanied by prominent astrogliosis (green, *iv*). Nuclei were counterstained with 4′,6-diamidino-2-phenylindole (DAPI) (blue, *iii* and *iv*). Scale bars: 500 μm (*i* and *ii*); 250 μm (*iii* and *iv*).

The studies described earlier indicate that the altered longevity-assurance activity of p53:p44 in p44^+/+^;APP_695/swe_ mice preferentially affects memory-forming areas of the brain. Therefore, we analyzed the association fibers of the corpus callosum, which are involved in memory retrieval and high-order activities requiring information transfer between the two hemispheres. Klüver–Barrera staining for simultaneous visualization of cell bodies and myelinated axon fibers revealed massive disorganization of the corpus callosum with loss of myelinated association fibers and prominent astrogliosis (Fig. 7).

Thus, the hippocampal formation and the corpus callosum represent the areas of the brain that were specifically and dramatically affected by the altered longevity-assurance activity of p53:p44 in APP_695/swe_ mice. Although the corpus callosum is contributed by cortical neurons, we did not observe widespread degeneration of the neocortex (see Fig. S4A) suggesting a very selective form of neurodegeneration. The above phenotype was evident under two different genetic backgrounds (see Experimental Procedures), in both males and females, and could be observed as early as 2.5 months of age before natural death of the animals.

**Fig. 8:**
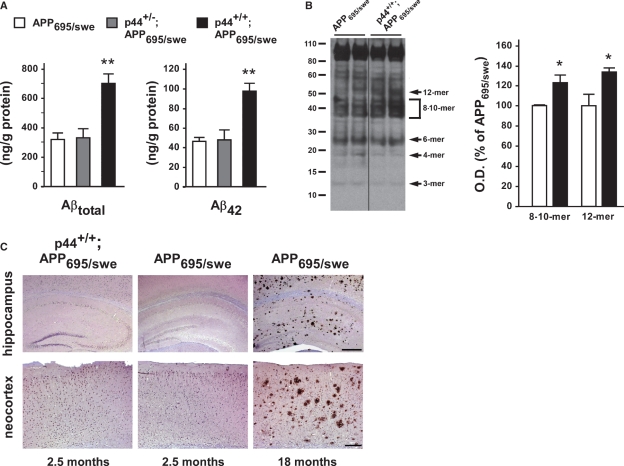
p44^+/+^;APP_695/swe_ transgenic mice have increased levels of Aβ but no amyloid plaques. (A) Soluble Aβ levels were measured by sandwich ELISA in brain homogenates (neocortex) of 2.5-month-old mice. p44^+/+^;APP_695/swe_ mice display a ∼2-fold increase in the levels of both Aβ_total_ and Aβ_42_. No difference was observed with the Aβ_42_/Aβ_total_ ratio. Similar results were obtained in the hippocampus (data not shown). (B) Aβ oligomers were immunoprecipitated from brain homogenates (neocortex) with antibody 6E10 and analyzed by Western blot. The ‘Aβ-bands’ shown here were identified with 6E10 antibody (against aa. 1-16 of the Aβ region of APP), but not with 22C11 (against the N-terminus of APP), indicating that they are neither degradation nor cleavage-end products of APP. In addition, the above bands were not detected with AB5352, which recognizes C-terminal fragments of APP. The molecular masses correspond to previously published and characterized Aβ oligomers (Lesne *et al.*, 2006). The 12-, 9-, 6-mer bands correspond to dodecameric (56-kDa), nonameric (40-kDa), and hexameric (27-kDa) Aβ aggregates. sAPP is also visible below the 110-kDa molecular marker. The graphic in the right panel shows relative optical density (OD) quantification of the indicated Aβ immunoreactive bands (expressed as percentage of APP_695/swe_ mice). Animals were 2.5-month old when analyzed. (C) Immunostaining of Aβ deposits in the hippocampus and neocortex of 2.5-month-old p44^+/+^;APP_695/swe_, age-matched APP_695/swe_, and 18-month-old APP_695/swe_ mice. Classical amyloid plaques were only observed in the old APP_695/swe_ animals. Scale bar (hippocampus): 500 μm. Scale bar (neocortex): 200 μm. All values are mean ± SEM. *P < 0.05, **P < 0.005.

We previously showed that hyperactivation of IGF-1R signaling results in increased levels of BACE1 and increased production of Aβ (Costantini *et al.*, 2006). Accordingly, the analysis of total brain homogenates revealed a ∼2-fold increase in Aβ levels in p44^+/+^;APP_695/swe_ mice when compared to APP_695/swe_ single-transgenics (Fig. 8A). Importantly, p44^+/−^;APP_695/swe_ littermates did not display increased production of Aβ (Fig. 8A). In fact, it is already known that IGF-1R hyperactivation, as caused by altered longevity-assurance activity of p53:p44, is only evident in homozygous animals. As a result, p44^+/−^ heterozygous mice have normal lifespan (Maier *et al.*, 2004). The increased production of Aβ in p44^+/+^;APP_695/swe_ double-transgenics was paralleled by increased levels of Aβ oligomers (Fig. 8B), although classical amyloid plaques were never observed in the young double-transgenics (Fig. 8C). Aβ small aggregates have been proposed as one of the possible causes of neuronal injury and synaptic damage in a variety of AD-like models (Klein *et al.*, 2001; Cleary *et al.*, 2005; Santacruz *et al.*, 2005; Lesne *et al.*, 2006; Cheng *et al.*, 2007). Importantly, we only detected increased levels of the high-molecular and neurotoxic Aβ species (> 8-mer), which mostly correspond to nonameric (∼40-kDa) and dodecameric (∼56-kDa; Aβ*56) aggregates (Fig. 8B). Although absent in 2.5-month-old p44^+/+^;APP_695/swe_ double-transgenics and APP_695/swe_ single-transgenics, amyloid plaques were evident in 18-month-old APP_695/swe_ mice (Fig. 8C), suggesting that the early death of the double-transgenic animals does not allow natural formation of plaques.

### The neurodegeneration in p44^+/+^;APP_695/swe_ mice is apoptosis-independent and appears to involve both paraptosis- and autophagy-like events

To investigate the mechanisms of the neuronal death, we initially analyzed the mRNA levels of common p53 pro-apoptotic targets, such as Puma, Noxa, and Bax. However, we did not observe any difference between p44^+/+^;APP_695/swe_ double-transgenics and their non-Tg littermates (data not shown). This finding is not completely surprising because the pro-apoptotic functions of p53 are linked to its tumor suppressor activity but not to its longevity-assurance activity [reviewed in (Bauer & Helfand, 2006; Rodier *et al.*, 2007; Ungewitter & Scrable, 2008)]. Consistently, we could not detect caspase activation, poly(ADP-ribose) polymerase (PARP) cleavage, or DNA fragmentation as assessed by TUNEL staining (data not shown). Therefore, in light of these results we had to rule out apoptosis as the main mechanism of neuronal death in our animals.

To further investigate the mechanisms of the cell death, we analyzed different areas of the hippocampal formation with transmission electron microscopy. We consistently observed two different types of cells, which display characteristics of two different types of programmed cell death. The first is represented by neurons that preserved their overall shape, did not display cellular blebbing, and maintained normal nuclei with smooth margins and no evidence of chromatin-clumping. These neurons appeared as lightly stained (see Fig. 9A; see also Fig. S9). Their cytoplasm displayed different collections of tightly packed cytoplasmic vacuoles, which often occupied a significant portion of the cell body. In some cases the vacuoles appeared as swollen and degenerating mitochondria; they were observed both inside the neuronal cell bodies and in the surrounding neuropil. In a few cases, the neurons were distorted by the presence of numerous large vacuoles (see Fig. S9). These ‘vacuolar-degenerating neurons’ co-existed with a second type of cells represented by darkly stained neurons (Fig. 9B; see also Fig. S9) displaying cell shrinkage, condensed nuclei, chromatin clustering, and ruffling of the plasma membrane. The chromatin clustering was different from the typical apoptosis-like condensation and appeared more like loose speckles and irregular peripheral bundles. The nuclear abnormalities included invagination and disruption of the nucleolemma (Fig. S9). In addition, these dark degenerating neurons displayed accumulation of lysosomes and dilated endoplasmic reticulum, Golgi apparatus, and mitochondria (Fig. 9B). The features displayed by the lightly stained and vacuolar-degenerating neurons are reminiscent of paraptosis, whereas those displayed by the darkly stained neurons are reminiscent of autophagic cell death. Both paraptosis and autophagy are caspase- and apoptosis-independent forms of cell death that have been previously described in *ex vivo* and *in vivo* settings [reviewed in (Broker *et al.*, 2005; Leist & Jaattela, 2001)]. Importantly, the presence of dark degenerating neurons has already been reported in both patients with AD (Nixon *et al.*, 2005) and AD-like mouse models (Yang *et al.*, 2008).

Paraptosis, as well as autophagic cell death, cannot be recognized with standardized *in vitro* biochemical assays. As a result, it is currently defined by its morphological features and by the absence of caspase activation or apoptosis-related markers [reviewed in (Broker *et al.*, 2005; Leist & Jaattela, 2001)]. However, when initially described in a cellular model of IGF-1R hyperactivation (Sperandio *et al.*, 2000), paraptosis appeared to be accompanied by activation of mitogen-activated protein kinases (MAPK) and Jun N-terminal kinases (JNK). As overexpression of p44 can cause hyperactivation of IGF-1R signaling [(Costantini *et al.*, 2006; Maier *et al.*, 2004); see also Fig. 2A], we investigated whether p44^+/+^;APP_695/swe_ mice displayed increased phosphorylation (activation) of MAPK and JNK. Analysis of hippocampal tissue of the double-transgenics revealed a significant increase in the phosphorylation of both JNK (p54 and p46) and MAPK (p44 and p42), when compared to non-Tg animals (Fig. 9C,D). Interestingly, p44^+/+^ single-transgenics also displayed increased phosphorylation of JNK and MAPK, although only JNK reached statistical significance (Fig. 9C,D). In addition to IGF-1R, paraptosis can also be induced by TAJ/TROY, a member of the tumor necrosis factor receptor superfamily (Wang *et al.*, 2004). However, we did not detect increased levels of TAJ/TROY or PDCD5, a downstream target of TAJ/TROY, in our animals (data not shown) excluding the involvement of this upstream effector.

When taken together, the above results indicate that the neurodegeneration observed in the brain of p44^+/+^;APP_695/swe_ double-transgenic animals is caspase- and apoptosis-independent; they also suggest that both paraptosis- and autophagy-like pathways might be involved, likely through the interplay of different signaling machineries that require interaction of IGF-1R with APP (or its proteolytic derivatives).

### *Igf1r* haploinsufficiency improves the synaptic deficits of APP_695/swe_ transgenic mice

**Fig. 10:**
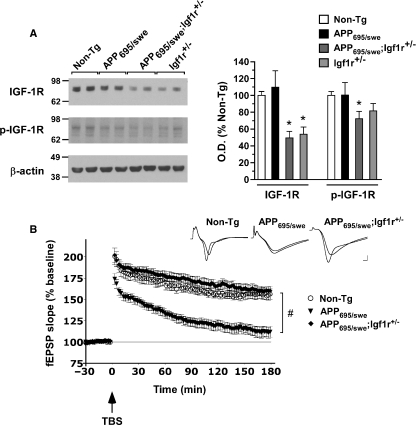
Down-regulation of IGF-1R improves the synaptic deficits of APP_695/swe_ animals. (A) Expression levels of IGF-1R and phospho-IGF-1R (p-IGF-1R) in the hippocampal formation of non-Tg, APP_695/swe_, APP_695/swe_;Igf1r^+/−^, and Igf1r^+/−^ animals. A representative Western blot of 2 different animals is shown in the left panel, whereas the percentage of change is shown in the right panel (*n*= 4). Animals were 2.5-month old when analyzed. (B) Enhanced hippocampal CA1 long-term potentiation (LTP) in APP_695/swe_;Igf1r^+/−^ transgenic animals. Theta burst stimulation (TBS)-induced LTP was enhanced in APP_695/swe_;Igf1r^+/−^ [*n* = 10(3)] compared to APP_695/swe_ animals [*n* = 13(3)]. Non-Tg mice [11(4)] are also shown. *Insets*: example traces before and after LTP for each transgenic group. Calibration 1 mV, 1 ms. No difference was observed in the paired-pulse facilitation (PPF) or i/o curves (data not shown). Animals were 2.5-month old when analyzed. All values are mean ± SEM. *P < 0.05; #*P* < 0.0005.

The results obtained with p44^+/+^;APP_695/swe_ double-transgenics indicate that hyperactivation of IGF-1R signaling, as caused by deregulated longevity-assurance activity of p53:p44, can cause/accelerate an AD-like neuropathology in the mouse. The fact that haploinsufficiency of the *Igf1r* gene can correct the synaptic deficits of p44^+/+^ single-transgenics prompted us to test whether IGF-1R is a valid pharmacologic target for the prevention/treatment of the synaptic deficits that occur in patients with AD. For this purpose, we generated APP_695/swe_;Igf1r^+/−^ double-transgenic mice and compared the synaptic functions of the single- and double-transgenics. Figure 10(A) shows that the double-transgenics displayed a significant decrease in the levels of both the total protein and the phosphorylated form of IGF-1R. LTP assessment of APP_695/swe_ mice revealed a marked defect in the late phase of LTP (Fig. 10B), in the absence of significant deficits in the presynaptic component of the synaptic transmission (data not shown). *Igf1r* haploinsufficiency in APP_695/swe_;Igf1r^+/−^ double-transgenics restored the LTP trace, thus proving that downregulation of IGF-1R signaling has positive effects even in the case of APP-driven pathology (Fig. 10B). While this manuscript was under preparation, Freude *et al.* (Freude *et al.*, 2009) reported that selective disruption of neuronal *Igf1r* reverts the AD-like pathology of Tg2576 mice, thus confirming our present results.

## Discussion

The implication of IGF-1R signaling in the regulation of aging and age-associated events has been consistently demonstrated in many organisms, including yeast, *C. elegans*, *D. melanogaster*, and mammals [reviewed in (Bauer & Helfand, 2006; Rodier *et al.*, 2007)]. Recent work has also revealed a possible mechanistic link between IGF-1R and age-associated neurodegenerative diseases [reviewed in (Cohen & Dillin, 2008; Puglielli, 2008)]. Importantly, genetic variations that cause reduced IGF-1R signaling appear to be beneficial for old age survival and for the preservation of cognitive functions in humans (van Heemst *et al.*, 2005; Suh *et al.*, 2008).

We previously published that hyperactivation of IGF-1R signaling, induced by the overexpression of Δ40p53 (p44), accelerates a naturally occurring and age-associated TrkA-to-p75^NTR^ switch in the brain that activates the lipid second messenger ceramide (Costantini *et al.*, 2005, 2006). Ceramide, in turn, regulates the steady-state levels of BACE1 and the production of Aβ (Costantini *et al.*, 2005, 2006). At the mechanistic level, ceramide controls a transient form of lysine acetylation of nascent BACE1 that impacts the cellular levels of the mature and enzymatically active β secretase (Costantini *et al.*, 2007; Jonas *et al.*, 2008; Ko & Puglielli, 2009). Here, we report that when in the presence of a ‘humanized’ version of mouse APP, the hyperactivation of IGF-1R signaling, as caused by altered p53:p44 longevity-assurance activity, results in massive and widespread degeneration of memory-forming and -retrieving areas of the brain. Mechanistically, the neurodegeneration of p44^+/+^;APP_695/swe_ double-transgenics was linked to a caspase- and apoptosis- independent form of cell death, specifically to a combination of paraptosis- and autophagy-like events. A caspase-driven form of neuronal death that follows the classical apoptosis pathway has been clearly and consistently documented in the developing brain. However, the evidence for a similar pathway in the adult brain is often limited [reviewed in (Leist & Jaattela, 2001)]. In fact, although some degree of classical caspase-dependent apoptosis can occur in the adult brain, mixed features of cell death are often observed. As a result, it is generally thought that at least part of the cell death in chronic neurodegenerative diseases is caused by alternative mechanisms [reviewed in (Leist & Jaattela, 2001)]. Autophagy can influence both cell survival and cell death (Nixon, 2006; Lee, 2009). In fact, a fully functioning autophagic system can help turn over organelles, cytoplasmic constituents, and toxic protein aggregates. However, when dysfunctional, autophagy can cause accumulation of autophagic vacuoles and trigger other forms of cell death (Nixon, 2006; Lee, 2009). Although the precise mechanisms of autophagy activation in neurodegenerative diseases are still being evaluated, it is now evident that, when dysfunctional, it represents an important form of cell death in both AD and non-AD forms of neurodegeneration (Nixon, 2006; Lee, 2009). Paraptosis was initially described in *ex vivo* settings as a result of hyperactivation of IGF-1R signaling (Sperandio *et al.*, 2000). Although it does not appear to have clearly recognizable biochemical markers, paraptosis has been linked to increased phosphorylation of the MAPK/JNK system, at least under conditions that involve IGF-1R activity (Sperandio *et al.*, 2004). Therefore, our results reinforce the idea that the phenotype of p44^+/+^;APP_695/swe_ mice depends heavily on the hyperactivation of IGF-1R signaling, which is caused by the altered longevity-assurance activity of the p53:p44 system. It is worth noting that the ability of IGF-1R to cause cell death has also been documented before, both in *ex vivo* and *in vivo* settings (Liu *et al.*, 1993, 1998; Plymate *et al.*, 1997; Hongo *et al.*, 1998). The fact that neither paraptosis nor autophagic cell death was observed in age-matched p44^+/+^ single-transgenics suggests that activation of the MAPK/JNK system alone, as a result of IGF-1R hyperactivation, is not enough to commit the neurons to death, at least *in vivo*. This is not entirely surprising as neurons differ from cultured cell lines and appear to possess alternative systems that ‘buffer’ pro-death signals [reviewed in (Leist & Jaattela, 2001)]. Therefore, it is likely that the ultimate commitment to death is caused by the interplay of different signaling machineries requiring, at least in our model, interaction of IGF-1R with APP (or its proteolytic derivatives). Further characterization and understanding of this novel form of cell death (paraptosis) and its apparent close relationship with autophagy in our double-transgenic mice might help us understand the events responsible for age-associated neurodegenerative diseases and open new perspectives for their treatment.

Both the hippocampal formation and the corpus callosum are affected very early in AD (Hyman *et al.*, 1984; Teipel *et al.*, 2003; Pogarell *et al.*, 2005) and play important roles in memory functions. In addition to AD, alterations of the corpus callosum are found in schizophrenia, chronic epilepsy, and autism (Paul *et al.*, 2007). Similarly, alterations of the afferent path together with abnormalities of the hippocampus are also observed in epilepsy (Kinney *et al.*, 2007; Auer *et al.*, 2008). Although the above disorders do not share all the other features that were observed in the double-transgenics, we did notice a slight propensity of the p44^+/+^;APP_695/swe_ animals to brief, spontaneous, and noninducible seizures, which did not appear to affect the normal life of the animals. Interestingly, hippocampal abnormalities have been identified in a subset of sudden unexplained death that occurs in otherwise normal children (Kinney *et al.*, 2007). The death appears to be caused by unwitnessed seizures arising during sleep in the anomalous hippocampus and causing cardiopulmonary arrest. In the absence of an evident cause of death at autopsy and ancillary testing, the above represents a viable explanation as the immediate cause of death of the double-transgenics. Importantly, it is already known that overexpression of p44^+/+^ in the mouse does not cause hypoglycemia or other metabolic abnormalities that could result in sudden death (Maier *et al.*, 2004). Finally, isolated seizures have been observed in other AD-like animal models (Westmark *et al.*, 2008) and are common in patients with advanced AD (Risse *et al.*, 1990; Menendez, 2005).

A previous report in aged rats suggested that plasma IGF-1 might enhance Aβ clearance from the brain (Carro *et al.*, 2002). However, subsequent studies in several animal models, which included APP-expressing mice, failed to detect changes in Aβ clearance, amyloid load, or in the phosphorylation status of tau, ruling out any disease-modifying benefits of an IGF-1 restorative therapy (Lanz *et al.*, 2008). Additionally, a recent study in patients with AD found no cognitive benefits when IGF-1 secretion in the plasma was induced with the growth hormone (GH) secretagogue MK-677 (Sevigny *et al.*, 2008). These results are not surprising. In fact, it is already known that brain IGF-1 is synthesized locally, is not under the control of GH, and is not affected by plasma/circulating IGF-1 [(Costantini *et al.*, 2006; Lupu *et al.*, 2001; Sun *et al.*, 2005), also reviewed in (Puglielli, 2008)]. Additionally, mutations that decrease the levels of IGF-1R or block IGF-1R signaling extend lifespan and improve age-associated manifestations, whereas hyperactivation of IGF-1R signaling produces the opposite effects [reviewed in (Bauer & Helfand, 2006; Kenyon, 2005; Puglielli, 2008; Rodier *et al.*, 2007)]. In the mouse, haploinsufficiency for *Igf-1r* or deletion of *insulin/IGF-1 receptor substrate 2* (*Irs2*), an essential component of IGF-1R signaling, leads to increased lifespan and improved cognitive/behavioral performance (Holzenberger *et al.*, 2003; Taguchi *et al.*, 2007). Recent work from Freude *et al.* (Freude *et al.*, 2009) showed that IRS2 deficiency in the Tg2576 AD mouse model reduced both the β cleavage of APP and the levels of Aβ in the brain and was able to reverse the premature mortality of the Tg2576 single-transgenics. Identical results were obtained when *Igf1r* was selectively disrupted in the neurons of Tg2576 animals (Freude *et al.*, 2009). It is also worth noting that studies in *C. elegans* indicate that IGF-1R signaling not only regulates longevity but also the cytotoxicity induced by protein aggregates (Cohen *et al.*, 2006; Cohen & Dillin, 2008). These results are consistent with our present findings and suggest that pharmacological targeting of IGF-1R should be explored for the treatment/prevention of both the cognitive decline that accompanies aging and the neuropathology that characterizes late-onset AD.

## Experimental procedures

### Transgenic mice

Animal experiments were carried out in accordance with the NIH Guide for the Care and Use of Laboratory Animals and were approved by the Institutional Animal Care and Use Committee of the University of Wisconsin-Madison and the Madison Veterans Administration Hospital. The animals described here were all littermates and under the same genetic background (ICR and mixed ICR × C57BL/6J). Details of breeding strategies and genotyping are described in Supporting Information. The generation of p44^+/+^ single-transgenics has been described before (Maier *et al.*, 2004). Transgenic mice heterozygous for a targeted disruption of *Ifg1r* gene (Igf1r^+/−^) were kindly provided by Dr. A. Efstratiadis (Columbia University, New York) (Liu *et al.*, 1993). APP_695/swe_ and Tau^−/−^ transgenic mice were from The Jackson Laboratory (Bar Harbor, Maine, USA). The normal husbandry of the mice has already been described (Maier *et al.*, 2004; Costantini *et al.*, 2005, 2006).

### Behavioral and cognitive testing

Animals were housed in less than four per cage, and all mice in a single cage were trained and tested at the same time. Mice were allowed to acclimate to the room for 30 min prior to training or testing. Only standard and widely used behavioral/cognitive tests (open field, fear conditioning to cue and context, and Barnes maze) were employed in this study. Details are described in Supporting Information.

### Electrophysiology

As described in the Supporting Information, 400 μm transverse hippocampal slices from 2.5-month-old mice were prepared. LTP was induced with theta burst stimulation applied to the Schaffer collaterals, at the border of CA3 and CA1, and field EPSPs were measured in stratum radiatum. Theta burst stimulation consisted of ten bursts/train, and three trains/stimulus with a 20-s intertrain interval. Each burst contained four stimulations at 100 Hz with an interburst interval of 200 ms. A detailed description of the procedure is found in the Supporting Information.

### Histology and immunostaining

Animals were euthanized according to the NIH Guide for the Care and Use of Laboratory Animals. The brains were dissected, fixed overnight in 10% neutral buffered formalin, and paraffin-embedded using standard techniques. Coronal tissue sections (5 μm) were prepared using a microtome. Following standard deparaffinization and rehydration, the tissue sections were processed for Hematoxylin and Eosin (H&E) staining or immunofluorescence. Antigen retrieval for immunofluorescent staining was performed in 100 mm of citrate buffer (pH 6) heated in an autoclave. After washing with PBS, tissue sections were permeabilized with 0.1% Triton X-100 in PBS and blocked for 2 h with 10% goat serum, 2% bovine serum albumin, and 0.1% Triton X-100 in PBS. Sections were then incubated with primary antibodies (diluted in blocking solution) overnight at 4 °C. After washing with PBS, they received fluorophore- or biotin-conjugated (anti-rabbit or anti-mouse) secondary antibodies for 1 h at room temperature. Nuclei were counterstained with 4′,6-diamidino-2-phenylindole (DAPI; Molecular Probes-Invitrogen, Carlsbad, CA, USA). Slides were mounted using Gel/Mount aqueous mounting medium (Electron Microscopy Sciences, Hatfield, PA, USA).

The following primary antibodies were used: rabbit anti-GFAP (polyclonal; 1:500; Dako, Carpinteria, CA, USA), mouse anti-NeuN (clone A60; 1:200; Chemicon-Millipore; Billerica, MA, USA), mouse anti-PHF-Tau (clone AT8; 10 μg/mL; Innogenetics-AutogenBioclear, Calne, UK), rabbit anti-phospho-S356-Tau (polyclonal; 1:100; Abcam; Cambridge, MA, USA), and rabbit anti-synaptophysin (monoclonal [YE269]; 1:250; Abcam). Secondary antibodies were Alexa 488- and Alexa 594-conjugated goat anti-rabbit (5 μg/mL; Molecular Probes-Invitrogen) for GFAP and synaptophysin, respectively. For NeuN and PHF-Tau-AT8 immunofluorescence, the secondary antibodies were biotin-labeled goat anti-mouse (5 μg/mL; Molecular Probes-Invitrogen) followed by Alexa 488- or Alexa 594-conjugated streptavidin (5 μg/mL; Molecular Probes-Invitrogen). Controls were performed by omitting the primary antibody. Kluver–Barrera method (Luxol Fast Blue-Cresyl Echt Violet; Poly Scientific, Bay Shore, NY, USA) for myelin and nerve cells was performed following the manufacturer’s instructions. Beta-amyloid staining was performed using Beta-Amyloid mouse monoclonal antibody (clone 6F/3D; 1/50; Novocastra, Newcastle upon Tyne, UK) after pretreatment of tissue sections with formic acid for 30 min.

Processed slides were imaged on a Zeiss Axiovert 200 inverted fluorescent microscope. Quantification of relative optical densities (ROD) of synaptophysin staining was performed in synaptophysin-immunolabeled coronal sections imaged at X20 using Scion-Image software. Measurements were performed in at least three brain sections for each animal. Mean ROD values were reported as percentage of non-Tg mice. Statistical analysis was performed using one-way anova followed by Tukey–Kramer multiple comparisons test. Differences were declared statistically significant if *P* < 0.05.

Timm’s sulfide silver staining was performed in 1.5-month-old male mice following the procedure described by Sloviter (Sloviter, 1982). Briefly, mice were perfused transcardially with a 0.37% sodium sulfide solution (phosphate-buffered) for 5 min, followed by 10% neutral-buffered formalin for additional 5 min. Brains were then removed, incubated overnight in formalin, and processed for paraffin embedding. Coronal tissue sections (10 μm) were incubated for 45 min in developer solution prepared immediately before use (Sloviter, 1982). Sections from all genotypes were processed together in the same rack. Development was stopped by incubation in tap water, and slides were counterstained with cresyl violet. Slides were then dehydrated and mounted with VectaMount Permanent Mounting Medium (Vector labs, Burlingame, CA, USA).

### Electron microscopy

Transmission electron microscopy was performed at the Electron Microscopy Facility of the University of Wisconsin-Madison, using 2.5% glutaraldehyde in phosphate buffer (pH 7.2) as a fixer (Oberley *et al.*, 2008). Tissues were rinsed in Sorenson’s phosphate washing buffer, postfixed in Caulfield’s osmium tetroxide plus sucrose, and rinsed again in washing buffer. All supplies and reagents were from Electron Microscopy Sciences (Hatfield, PA, USA). Tissues were dehydrated in a graded series of ethanol and propylene oxide and embedded in BEEM™ capsules using EMbed-812 resin. Polymerization was thermally induced overnight in a 60 °C oven. Thin sections (70–80 nm) were cut and mounted on copper grids, stained with filtered 7.7% uranyl acetate and counterstained with Reynolds lead citrate. Tissue sections were observed using a transmission electron microscope (Hitachi H-600) operated at 75 kV.

### Western blot analysis

Protein extracts from the hippocampus of 2.5-month-old transgenic and nontransgenic littermates were prepared in GTIP buffer (100 mm Tris pH 7.6, 20 mm EDTA, 1.5 m NaCl) supplemented with 1% Triton X-100 (Roche, Indianapolis, IN, USA), 0.25% NP40 (Roche), complete protein inhibitors cocktail (Roche), and phosphatases inhibitors (cocktail set I and set II; Calbiochem-EMD, San Diego, CA, USA). Protein concentration was measured by the bicinchoninic acid method (Pierce-Thermo Scientific, Rockford, IL, USA). Protein samples (20 or 40 μg) prepared in reducing NuPAGE® LDS Sample Buffer (Invitrogen) were subjected to electrophoresis using precast NuPAGE® Novex 4–12% Bis-Tris gels (Invitrogen) and transferred to nitrocellulose membranes (Invitrogen). Membranes were blocked for 1 h in Tris-buffered saline–0.1% Tween®20 (TBST) containing 5% nonfat dry milk, followed by an overnight incubation with primary antibody diluted in 5% BSA in TBST. After washing with TBST, the membrane was incubated with peroxidase-conjugated secondary antibody (Amersham Biosciences-GE Healthcare, Piscataway, NJ, USA) for 1 h and developed using the LumiGLO chemiluminescent detection system (KPL; Gaithersburg, MD, USA). The following primary antibodies were used: anti-IGF-1 Receptor β (111A9) (1:1000; Cell Signaling, Danvers, MA, USA), anti-phospho-IGF-1 Receptor (Tyr1135/1136)/Insulin Receptor (Tyr1150/1151) (19H7) (1:1000; Cell Signaling), anti-tau (TAU-5) (1:1000; Invitrogen-BioSource™), anti-β-actin (1:1000; Cell Siganling), anti-SAPK/JNK (1:1000; Cell Signaling), anti-phospho-SAPK/JNK (Thr183/Tyr185) (1:1000; Cell Signaling), anti-p44/42 MAPK (137F5) (1:1000; Cell Signaling), anti-phospho-p44/42 MAPK (Erk1/2) (Thr202/Tyr204) (D13.14.4E) (1:2000; Cell Signaling). Densitometric analysis was performed using the NIH Image program.

### Aβ determination

Aβ levels were determined by standard sandwich ELISA using Beta-Amyloid 1–40 and 1–42 ELISA kits from Signet Laboratories (Covance, Princeton, NJ, USA) according to the protocol provided by the manufacturer. Samples were assayed by triplicate, and the levels of Aβ40, Aβ42, and Aβtotal were determined based upon standard curves run on every ELISA plate and then expressed as ng Aβ/g total protein.

### Immunoprecipitation and Western blot for Aβ oligomers

Forebrain protein extracts from 2.5-month-old APP_695/swe_ and double-transgenic p44^+/+^;APP_695/swe_ mice were prepared as described earlier. Immunoprecipitation was performed using 6E10 monoclonal antibody (against Aβ_1–16_; 1/100, Signet-Covance, Berkeley, CA, USA) and ExactaCruz™ E immunoprecipitation kit (Santa Cruz Biotechnolgy; Santa Cruz, CA, USA), to eliminate IP antibody detection in the subsequent Western blot analysis. Briefly, the protein extracts (500 μg diluted to 1 μg/μL) were precleared with Preclearing Matrix E for 30 min at 4 °C, transferred to the 6E10 antibody-IP matrix complex, and incubated overnight at 4 °C with slow rotation. After washing, pelleted matrix was resuspended in 30 μL of 2× reducing NuPAGE® LDS Sample Buffer (Invitrogen) and boiled for 5 min. Samples were subjected to electrophoresis using precast NuPAGE® Novex 4–12% Bis-Tris gels (Invitrogen) and transferred to nitrocellulose membranes (Invitrogen). Membranes were boiled for 5 min in PBS, blocked in Tris-buffered saline–0.1% Tween®20 (TBST) for 1 h and incubated for 2 h with 6E10 monoclonal antibody (1/200) diluted in ExactaCruz E dilution buffer. After washing with TBST, membranes were incubated for 1 h with ExactaCruz E Western Blot Reagent (1/1000) and then washed and developed as described earlier. After stripping, membranes were reprobed using monoclonal 22C11 (Chemicon-Millipore) or polyclonal APP C-terminus antibodies (Chemicon-Millipore).

### Statistical analysis

Data analysis was performed using GraphPad InStat 3.06 statistical software (GraphPad Software, San Diego, CA, USA). Data is expressed as mean ± SEM. Comparison of the means was performed using t-tests or one-way anova followed by Tukey–Kramer multiple comparisons test, unless otherwise noted. For lifespan assessment, data were analyzed with the Kaplan–Meier lifespan test and log-rank test using GraphPad Prism version 4.0 (GraphPad Software). Differences were declared statistically significant if *P* ≤ 0.05.

## Supporting Information

**Fig. S1** Open-field test of p44^+/+^ transgenic mice.

**Fig. S2** Short-term facilitation and basal synaptic transmission in p44^+/+^ mice.

**Fig. S3** Histology of peripheral organs from non-Tg and p44^+/+^;APP_695/swe_ double-transgenic mice.

**Fig. S4** Histology of neocortex, cerebellum, and spinal cord of non-Tg and p44^+/+^;APP_695/swe_ double-transgenic mice.

**Fig. S5** Histology of the hippocampus and dentate gyrus of APP_695/swe_, p44^+/−^, p44^+/−^;APP_695/swe_, and p44^+/+^ animals.

**Fig. S6** Widespread astrogliosis in the hippocampus and dentate gyrus of p44^+/+^;APP_695/swe_ transgenic mice.

**Fig. S7** Abnormal phosphorylation of the microtubule-associated protein tau in the hippocampus and dentate gyrus of p44^+/+^;APP_695/swe_ transgenic mice.

**Fig. S8** Loss of Timm’s staining in p44^+/+^;APP_695/swe_ double-transgenic mice.

**Fig. S9** Evidence of paraptosis- and autophagy-like cell death in the hippocampus of p44^+/+^;APP_695/swe_ double-transgenic mice.

As a service to our authors and readers, this journal provides supporting information supplied by the authors. Such materials are peer-reviewed and may be re-organized for online delivery, but are not copy-edited or typeset. Technical support issues arising from supporting information (other than missing files) should be addressed to the authors.

## References

[b1] Auer T, Barsi P, Bone B, Angyalosi A, Aradi M, Szalay C, Horvath RA, Kovacs N, Kotek G, Fogarasi A, Komoly S, Janszky I, Schwarcz A, Janszky J (2008). History of simple febrile seizures is associated with hippocampal abnormalities in adults. Epilepsia.

[b2] Bancher C, Brunner C, Lassmann H, Budka H, Jellinger K, Wiche G, Seitelberger F, Grundke-Iqbal I, Iqbal K, Wisniewski HM (1989). Accumulation of abnormally phosphorylated tau precedes the formation of neurofibrillary tangles in Alzheimer’s disease. Brain Res..

[b3] Bauer JH, Helfand SL (2006). New tricks of an old molecule: lifespan regulation by p53. Aging Cell.

[b4] Borchelt DR, Thinakaran G, Eckman CB, Lee MK, Davenport F, Ratovitsky T, Prada CM, Kim G, Seekins S, Yager D, Slunt HH, Wang R, Seeger M, Levey AI, Gandy SE, Copeland NG, Jenkins NA, Price DL, Younkin SG, Sisodia SS (1996). Familial Alzheimer’s disease-linked presenilin 1 variants elevate Abeta1-42/1-40 ratio in vitro and in vivo. Neuron.

[b5] Bourdon JC, Fernandes K, Murray-Zmijewski F, Liu G, Diot A, Xirodimas DP, Saville MK, Lane DP (2005). p53 isoforms can regulate p53 transcriptional activity. Genes Dev..

[b6] Broker LE, Kruyt FA, Giaccone G (2005). Cell death independent of caspases: a review. Clin. Cancer Res..

[b7] Carro E, Trejo JL, Gomez-Isla T, LeRoith D, Torres-Aleman I (2002). Serum insulin-like growth factor I regulates brain amyloid-beta levels. Nat. Med..

[b8] Cheng IH, Scearce-Levie K, Legleiter J, Palop JJ, Gerstein H, Bien-Ly N, Puolivali J, Lesne S, Ashe KH, Muchowski PJ, Mucke L (2007). Accelerating amyloid-beta fibrillization reduces oligomer levels and functional deficits in Alzheimer disease mouse models. J. Biol. Chem..

[b9] Cleary JP, Walsh DM, Hofmeister JJ, Shankar GM, Kuskowski MA, Selkoe DJ, Ashe KH (2005). Natural oligomers of the amyloid-beta protein specifically disrupt cognitive function. Nat. Neurosci..

[b10] Cohen E, Dillin A (2008). The insulin paradox: aging, proteotoxicity and neurodegeneration. Nat. Rev. Neurosci..

[b11] Cohen E, Bieschke J, Perciavalle RM, Kelly JW, Dillin A (2006). Opposing activities protect against age-onset proteotoxicity. Science.

[b12] Costantini C, Weindruch R, Della Valle G, Puglielli L (2005). A TrkA-to-p75NTR molecular switch activates amyloid beta-peptide generation during aging. Biochem. J..

[b13] Costantini C, Scrable H, Puglielli L (2006). An aging pathway controls the TrkA to p75(NTR) receptor switch and amyloid beta-peptide generation. EMBO J..

[b14] Costantini C, Ko MH, Jonas MC, Puglielli L (2007). A reversible form of lysine acetylation in the ER and Golgi lumen controls the molecular stabilization of BACE1. Biochem. J..

[b15] Courtois S, Verhaegh G, North S, Luciani MG, Lassus P, Hibner U, Oren M, Hainaut P (2002). DeltaN-p53, a natural isoform of p53 lacking the first transactivation domain, counteracts growth suppression by wild-type p53. Oncogene.

[b16] Duyckaerts C, Potier MC, Delatour B (2008). Alzheimer disease models and human neuropathology: similarities and differences. Acta Neuropathol..

[b17] Freude S, Hettich MM, Schumann C, Stohr O, Koch L, Kohler C, Udelhoven M, Leeser U, Muller M, Kubota N, Kadowaki T, Krone W, Schroder H, Bruning JC, Schubert M (2009). Neuronal IGF-1 resistance reduces A{beta} accumulation and protects against premature death in a model of Alzheimer’s disease. FASEB J..

[b18] Ghosh A, Stewart D, Matlashewski G (2004). Regulation of human p53 activity and cell localization by alternative splicing. Mol. Cell. Biol..

[b19] Hedden T, Gabrieli JD (2005). Healthy and pathological processes in adult development: new evidence from neuroimaging of the aging brain. Curr. Opin. Neurol..

[b20] van Heemst D, Beekman M, Mooijaart SP, Heijmans BT, Brandt BW, Zwaan BJ, Slagboom PE, Westendorp RG (2005). Reduced insulin/IGF-1 signalling and human longevity. Aging Cell.

[b21] Holzenberger M, Dupont J, Ducos B, Leneuve P, Geloen A, Even PC, Cervera P, Le Bouc Y (2003). IGF-1 receptor regulates lifespan and resistance to oxidative stress in mice. Nature.

[b22] Hongo A, Yumet G, Resnicoff M, Romano G, O’Connor R, Baserga R (1998). Inhibition of tumorigenesis and induction of apoptosis in human tumor cells by the stable expression of a myristylated COOH terminus of the insulin-like growth factor I receptor. Cancer Res..

[b23] Hyman BT, Van Hoesen GW, Damasio AR, Barnes CL (1984). Alzheimer’s disease: cell-specific pathology isolates the hippocampal formation. Science.

[b24] Jonas MC, Costantini C, Puglielli L (2008). PCSK9 is required for the disposal of non-acetylated intermediates of the nascent membrane protein BACE1. EMBO Rep..

[b25] Kenyon C (2005). The plasticity of aging: insights from long-lived mutants. Cell.

[b26] Kinney HC, Armstrong DL, Chadwick AE, Crandall LA, Hilbert C, Belliveau RA, Kupsky WJ, Krous HF (2007). Sudden death in toddlers associated with developmental abnormalities of the hippocampus: a report of five cases. Pediatr. Dev. Pathol..

[b27] Klein WL, Krafft GA, Finch CE (2001). Targeting small Abeta oligomers: the solution to an Alzheimer’s disease conundrum?. Trends Neurosci..

[b28] Ko MH, Puglielli L (2009). Two endoplasmic reticulum (ER)/ER golgi intermediate compartment-based lysine acetyltransferases post-translationally regulate BACE1 levels. J. Biol. Chem..

[b29] Lanz TA, Salatto CT, Semproni AR, Marconi M, Brown TM, Richter KE, Schmidt K, Nelson FR, Schachter JB (2008). Peripheral elevation of IGF-1 fails to alter Abeta clearance in multiple in vivo models. Biochem. Pharmacol..

[b30] Lee JA (2009). Autophagy in neurodegeneration: two sides of the same coin. BMB Rep..

[b31] Lee VM, Goedert M, Trojanowski JQ (2001). Neurodegenerative tauopathies. Annu. Rev. Neurosci..

[b32] Leist M, Jaattela M (2001). Four deaths and a funeral: from caspases to alternative mechanisms. Nat. Rev. Mol. Cell Biol..

[b33] Lesne S, Koh MT, Kotilinek L, Kayed R, Glabe CG, Yang A, Gallagher M, Ashe KH (2006). A specific amyloid-beta protein assembly in the brain impairs memory. Nature.

[b34] Liu JP, Baker J, Perkins AS, Robertson EJ, Efstratiadis A (1993). Mice carrying null mutations of the genes encoding insulin-like growth factor I (Igf-1) and type 1 IGF receptor (Igf1r). Cell.

[b35] Liu Y, Lehar S, Corvi C, Payne G, O’Connor R (1998). Expression of the insulin-like growth factor I receptor C terminus as a myristylated protein leads to induction of apoptosis in tumor cells. Cancer Res..

[b36] Lupu F, Terwilliger JD, Lee K, Segre GV, Efstratiadis A (2001). Roles of growth hormone and insulin-like growth factor 1 in mouse postnatal growth. Dev. Biol..

[b37] Maier B, Gluba W, Bernier B, Turner T, Mohammad K, Guise T, Sutherland A, Thorner M, Scrable H (2004). Modulation of mammalian life span by the short isoform of p53. Genes Dev..

[b38] Menendez M (2005). Down syndrome, Alzheimer’s disease and seizures. Brain Dev..

[b39] Nixon RA (2006). Autophagy in neurodegenerative disease: friend, foe or turncoat?. Trends Neurosci..

[b40] Nixon RA, Wegiel J, Kumar A, Yu WH, Peterhoff C, Cataldo A, Cuervo AM (2005). Extensive involvement of autophagy in Alzheimer disease: an immuno-electron microscopy study. J. Neuropathol. Exp. Neurol..

[b41] Oberley TD, Swanlund JM, Zhang HJ, Kregel KC (2008). Aging results in increased autophagy of mitochondria and protein nitration in rat hepatocytes following heat stress. J. Histochem. Cytochem..

[b42] Paul LK, Brown WS, Adolphs R, Tyszka JM, Richards LJ, Mukherjee P, Sherr EH (2007). Agenesis of the corpus callosum: genetic, developmental and functional aspects of connectivity. Nat. Rev. Neurosci..

[b43] Plymate SS, Bae VL, Maddison L, Quinn LS, Ware JL (1997). Type-1 insulin-like growth factor receptor reexpression in the malignant phenotype of SV40-T-immortalized human prostate epithelial cells enhances apoptosis. Endocrine.

[b44] Pogarell O, Teipel SJ, Juckel G, Gootjes L, Moller T, Burger K, Leinsinger G, Moller HJ, Hegerl U, Hampel H (2005). EEG coherence reflects regional corpus callosum area in Alzheimer’s disease. J. Neurol. Neurosurg. Psychiatry.

[b45] Puglielli L (2008). Aging of the brain, neurotrophin signaling, and Alzheimer’s disease: is IGF1-R the common culprit?. Neurobiol. Aging.

[b46] Risse SC, Lampe TH, Bird TD, Nochlin D, Sumi SM, Keenan T, Cubberley L, Peskind E, Raskind MA (1990). Myoclonus, seizures, and paratonia in Alzheimer disease. Alzheimer Dis. Assoc. Disord..

[b47] Rodier F, Campisi J, Bhaumik D (2007). Two faces of p53: aging and tumor suppression. Nucleic Acids Res..

[b48] Santacruz K, Lewis J, Spires T, Paulson J, Kotilinek L, Ingelsson M, Guimaraes A, DeTure M, Ramsden M, McGowan E, Forster C, Yue M, Orne J, Janus C, Mariash A, Kuskowski M, Hyman B, Hutton M, Ashe KH (2005). Tau suppression in a neurodegenerative mouse model improves memory function. Science.

[b49] Savonenko AV, Xu GM, Price DL, Borchelt DR, Markowska AL (2003). Normal cognitive behavior in two distinct congenic lines of transgenic mice hyperexpressing mutant APP SWE. Neurobiol. Dis..

[b50] Sevigny JJ, Ryan JM, van Dyck CH, Peng Y, Lines CR, Nessly ML (2008). Growth hormone secretagogue MK-677: no clinical effect on AD progression in a randomized trial. Neurology.

[b51] Sloviter RS (1982). A simplified Timm stain procedure compatible with formaldehyde fixation and routine paraffin embedding of rat brain. Brain Res. Bull..

[b52] Sperandio S, de Belle I, Bredesen DE (2000). An alternative, nonapoptotic form of programmed cell death. Proc. Natl Acad. Sci. USA.

[b53] Sperandio S, Poksay K, de Belle I, Lafuente MJ, Liu B, Nasir J, Bredesen DE (2004). Paraptosis: mediation by MAP kinases and inhibition by AIP-1/Alix. Cell Death Differ..

[b54] Suh Y, Atzmon G, Cho MO, Hwang D, Liu B, Leahy DJ, Barzilai N, Cohen P (2008). Functionally significant insulin-like growth factor I receptor mutations in centenarians. Proc. Natl Acad. Sci. USA.

[b55] Sun LY, Al-Regaiey K, Masternak MM, Wang J, Bartke A (2005). Local expression of GH and IGF-1 in the hippocampus of GH-deficient long-lived mice. Neurobiol. Aging.

[b56] Taguchi A, Wartschow LM, White MF (2007). Brain IRS2 signaling coordinates life span and nutrient homeostasis. Science.

[b57] Teipel SJ, Bayer W, Alexander GE, Bokde AL, Zebuhr Y, Teichberg D, Muller-Spahn F, Schapiro MB, Moller HJ, Rapoport SI, Hampel H (2003). Regional pattern of hippocampus and corpus callosum atrophy in Alzheimer’s disease in relation to dementia severity: evidence for early neocortical degeneration. Neurobiol. Aging.

[b58] Tissenbaum HA, Guarente L (2002). Model organisms as a guide to mammalian aging. Dev Cell.

[b59] Tyner SD, Venkatachalam S, Choi J, Jones S, Ghebranious N, Igelmann H, Lu X, Soron G, Cooper B, Brayton C, Hee Park S, Thompson T, Karsenty G, Bradley A, Donehower LA (2002). p53 mutant mice that display early ageing-associated phenotypes. Nature.

[b60] Ungewitter E, Scrable H (2008). Antagonistic pleiotropy and p53. Mech. Ageing Dev..

[b61] Wang Y, Li X, Wang L, Ding P, Zhang Y, Han W, Ma D (2004). An alternative form of paraptosis-like cell death, triggered by TAJ/TROY and enhanced by PDCD5 overexpression. J. Cell Sci..

[b62] Westmark CJ, Westmark PR, Beard AM, Hildebrandt SM, Malter JS (2008). Seizure susceptibility and mortality in mice that over-express amyloid precursor protein. Int. J. Clin. Exp. Pathol..

[b63] Yang DS, Kumar A, Stavrides P, Peterson J, Peterhoff CM, Pawlik M, Levy E, Cataldo AM, Nixon RA (2008). Neuronal apoptosis and autophagy cross talk in aging PS/APP mice, a model of Alzheimer’s disease. Am. J. Pathol..

[b64] Yankner BA, Lu T, Loerch P (2008). The aging brain. Annu. Rev. Pathol..

[b65] Yin Y, Stephen CW, Luciani MG, Fahraeus R (2002). p53 Stability and activity is regulated by Mdm2-mediated induction of alternative p53 translation products. Nat. Cell Biol..

