# Supplementary material for: Altered longevity-assurance activity of p53:p44 in the mouse causes memory loss, neurodegeneration and premature death
Source: Aging Cell. 2010 Apr;9(2):174–90. doi: 10.1111/j.1474-9726.2010.00547.x (PMC2848983; doi:10.1111/j.1474-9726.2010.00547.x)
Supplement: Supplementary file 1 [file ace0009-0174-SD1.pdf]

SUPPORTING INFORMATION

ALTERED LONGEVITY-ASSURANCE ACTIVITY OF P53:P44 IN THE MOUSE CAUSES MEMORY LOSS, NEURODEGENERATION AND PREMATURE DEATH

Mariana Pehar, Kenneth J. O'Riordan, Melissa Burns-Cusato, Matthew E. Andrzejewski, Carlos Gil del Alcazar, Corinna Burger, Heidi Scrable, and Luigi Puglielli

SUPPLEMENTARY EXPERIMENTAL PROCEDURES

Transgenic mice

Double-transgenic $p44^{+/+}$;$APP_{695/swe}$ mice were obtained by breeding hemizygous $APP_{695/swe}$ mice on a C57BL/6J background (B6.Cg-Tg(Prnp-App/APP_{swe})E1-2Dbo/J; The Jackson Laboratory, Bar Harbor, Maine, USA) with homozygous $p44^{+/+}$ mice on an ICR background (Maier *et al.*, 2004). $APP_{695/swe}$ mice express a chimeric mouse amyloid precursor protein (APP) cDNA encoding the 695-amino acid isoform with a "humanized" Aβ domain amino acid sequence that includes the familial AD-associated Swedish double mutation (K595N and M596L).

The $p44^{+/+}$ x $APP_{695/swe}$ cross yielded $p44^{+/-}$ and $p44^{+/-}$;$APP_{695/swe}$ animals on a mixed ICR x C57BL/6J background. $p44^{+/-}$;$APP_{695/swe}$ mice were then bred for at least 5 generations with homozygous $p44^{+/+}$ (ICR) mice to generate offspring on an ICR background. $p44^{+/-}$;$APP_{695/swe}$ (ICR) mice were bred with $p44^{+/-}$ (ICR) to generate transgenic ($p44^{+/-}$, $p44^{+/+}$, $APP_{695/swe}$, $p44^{+/-}$;$APP_{695/swe}$, and $p44^{+/+}$;$APP_{695/swe}$) and non-transgenic (Non-Tg) littermates. All the experiments reported here were done with the above animals on an ICR background. However, we also generated transgenics on a mixed ICR x C57BL/6J genetic background by breeding $p44^{+/-}$ (F1) with $p44^{+/-}$;$APP_{695/swe}$ (F1) and obtained identical results. Genotypes were determined shortly after weaning. The presence of the *App* transgene was determined as recommended by The Jackson Laboratory, whereas the presence of the Δ40p53 (p44) transgene was determined by PCR using the following specific primers: 5'-AAGCCTCGAAGTAAGTTGGATGCCTAG-3' and 5'-TGGCGGGATGTATCTTAAACCTTCC-3' (289bp product).

The results described in this work with the above $p44^{+/+}$;$APP_{695/swe}$ animals were also observed when $p44^{+/+}$ mice were crossed with Tg2576 expressing the entire human $APP_{695/swe}$ amino acid isoform (Hsiao *et al.*, 1996; Irizarry *et al.*, 1997; Lesne *et al.*, 2006). Tg2576 mice were a generous gift of Dr. James Malter.

Double-transgenic $p44^{+/+}$;$Igf1r^{+/-}$ mice were obtained by breeding heterozygous $Igf1r^{+/-}$ mice (Liu *et al.*, 1993) with homozygous $p44^{+/+}$ mice (Maier *et al.*, 2004). The presence of the Igf1r transgenic allele was determined by PCR using specific primers described in (Liu *et al.*, 1993). Double-transgenic $p44^{+/+}$;$Tau^{+/-}$ mice were obtained by

breeding homozygous Tau$^{-/-}$ mice (*Mapt*$^{tm1(EGFP)Klt}$/J; The Jackson Laboratory) with homozygous p44$^{+/+}$ mice (Maier *et al.*, 2004). The presence of the transgenic allele was determined by PCR as indicated by The Jackson Laboratory.

The normal husbandry of the mice has already been described (Costantini *et al.*, 2006; Costantini *et al.*, 2005; Maier *et al.*, 2004).

Behavioral Testing

Open Field Test

Basal locomotor activity levels and anxiety were assessed with the open field task. The open field arena, a square opaque plexiglass box (37x37x20 cm^2), was cleaned with 30% EtOH prior to each session. Each mouse was placed in the center of the open field arena alone and spontaneous activity was recorded for 15 minutes using an automated video-based system, configured to capture 4 images per second (4 hz) (Limelight, Coulbourn Instruments, Whitehall, PA, USA). Total distance traveled was used as a measure of activity and the center:total distance ratio was used as an index of anxiety.

Fear Conditioning

Associative memories were assessed using a delay fear conditioning protocol conducted in four identical commercially constructed chambers (18x18x 28 cm) coupled to an automated video-based tracking system (Coulbourn Instruments, Whitehall, PA, USA). Each of the chamber/camera units were housed in individual sheet vinyl sound- and light-attenuating cubicles. Prior to individual training/testing sessions, each chamber was cleaned with 30% EtOH (30% Isopropyl alcohol for cue testing). All the events in the protocol were programmed and data recorded through FreezeFrame2 and FreezeView software from Actimetrics Software (Wilmette, IL, USA). The training protocol used was a 6 minute long, 2-trial delay conditioning procedure. Mice were placed in the chambers with the houselight on. After 2 min, a 92 dB white noise stimulus (conditional stimulus, CS) was played for 30 sec; 28.5 sec after onset of the CS, a 1.5 sec. 0.5 mA shock (unconditional stimulus, US) was delivered to each floor. This was followed by a 2 min inter-trial-interval, during which only the houselight was on. Then, the white noise CS was again played for 30 sec, co-terminating with a 1.5 sec US. This was followed by a 60 sec period during which only the houselight was on. For *Context Testing*, 24hs after

training, mice were placed back into the same chamber in which they received the conditioning session. The houselight was on for the entirety of the session (5 min), but no stimuli were presented (e.g., no CS or US). Two hours after context-testing, mice were tested for conditioning to the CS ("cue"). The chamber was changed in several ways to provide a novel environment. The *cue testing* session was identical to the training session except that the US was not presented. Percent of time freezing to context was defined as the percent time freezing in the 5 minute context test *minus* percent time baseline freezing (measured during the first 2 min of the training session, before exposure to either CS or US). Percent time freezing to cue was defined as the percent time freezing to the CS presentations during cue test *minus* percent time freezing to newly configured context (computed during the first 2 min of the cue testing session). All data were analyzed using ANOVA, followed by protected *t*-tests comparing separate genotypes to non-transgenic controls.

Barnes Maze

Spatial learning was assessed by using the Barnes maze, a task based on the preference of mice for dark enclosed areas over bright open platforms. The maze consists of a circular white plastic table (122 cm diameter, 91.44 cm from floor). The periphery of the tabletop is lined with 20 evenly spaced holes and beneath each hole there is a small compartment constructed of black plexiglass. Only one of the black compartments is large enough for the mouse to enter completely. This compartment constitutes the goal (escape box), which is identified using visual extra-maze cues. A white PVC cylinder (20.3 cm diameter, 25.4 cm high) placed in the center of the maze constituted the start box. The experiment began with a habituation session, in which the start box was placed over the hole that leads to the goal box. The mouse was placed inside the start box and observed until it entered the goal box, in which it was allowed to remain for 2 min before being removed. This process was repeated for a total of 3 trials. The experimental training was conducted the following day. During this training the goal box was placed under a different hole from habituation and its position was changed between different subjects. The trial started with the start box located in the center of the maze and the individual subject placed inside it. After 30 sec, the start box was lifted and the mouse was free to explore the maze until it entered the goal box or until 3 min elapsed. If the

subject entered the goal box, it was allowed to stay there for 2 min. If the mouse did not find or enter the goal box, it was gently guided to the goal box and allowed to remain there for 2 min. The mouse was then returned to the home cage and the start box, goal box, and platform were thoroughly cleaned with an ethanol solution to prevent use of odor-cues for navigation on subsequent trials. A total of 4 trials, with 15 min inter-trial-interval, were conducted daily for 4 consecutively days. Each trial was video recorded and then analyzed with Noldus Ethovision software (Leesburg, VA, USA). Number of errors (sniffing over an incorrect hole) and latency to enter the goal box was recorded for all subjects. Distance traveled from start position to goal box was recorded for only 6 subjects per genotype due to technical constraints. Data from the last trial (trial 4) of each of the four training days were analyzed by ANOVA using mixed analysis of variance, with genotype as a between-subjects variable and training day as a within-subjects variable.

Electrophysiology

Hippocampal slices (400 μM) from 2.5 months old mice were prepared using a vibratome and submerged in ice cold cutting saline (CS; 110 mM Sucrose, 60 mM NaCl, 3 mM KCl, 1.25 mM NaH2PO4, 28 mM NaHCO3, 0.5 mM CaCl2, 7 mM MgCl2, 5 mM glucose, 0.6 mM ascorbate). Slices were allowed to recover for 45 min at room temperature (RT) in 50:50 CS:Artificial Cerebro-Spinal Fluid (ACSF; 125 mM NaCl, 2.5 mM KCl, 1.25 mM NaH2PO4, 25 mM NaHCO3, 2 mM CaCl2, 1 mM MgCl2, 25 mM glucose) and further incubated for 45 min at RT in 100% ACSF, before being transferred to an interface chamber (Fine Science Tools, Foster City, CA, USA) bathed in 100% ACSF (1 mL/min) at 32°C (TC-324B, Warner Instrument Corporation, Hamden, CT, USA) for 2 hours prior to experimentation. All solutions were carb-oxygenated (95/5, O_2/CO_2). Enameled bipolar platinum-tungsten (92:8 Pt:Y) stimulating electrodes were placed at the border of Area CA3 and Area CA1 along the Schaffer-collateral pathway. Field EPSPs were recorded from CA1 *stratum radiatum*, with ACSF filled recording electrodes (5 MΩ). Baseline synaptic transmission was assessed for each individual slice by applying gradually increasing stimuli (0.5V – 15V, 25nA – 1.5μA, A-M Systems model 2200 stimulus isolator, Carlsborg, WA, USA) to determine the input:output

relationship. All subsequent experimental stimuli were 50% of the intensity of the maximum evoked fEPSP slope. Paired-pulse facilitation (PPF) was investigated at various interstimulus intervals (20, 50, 100, 200, and 300 msec). LTP was induced with theta burst stimulation to the Schaffer-collaterals, at the border of CA3 and CA1, and field EPSPs were measured in stratum radiatum. Theta burst stimulation consisted of 10 bursts/train, and 3 trains/stimulus with a 20 sec intertrain interval. Each burst contained 4 stimulations at 100 Hz with an interburst interval of 200 msec. Synaptic efficacy was continually monitored (0.05 Hz). Every 2 min sweeps were averaged; the fEPSP's were amplified (A-M Systems model 1800), digitized (Digidata 1322B, Molecular Devices, Sunnyvale, CA, USA) and then analyzed (pClamp, Molecular Devices). Two-way ANOVA with repeated measures (mixed model) and Bonferroni post-tests were used for statistical analysis.

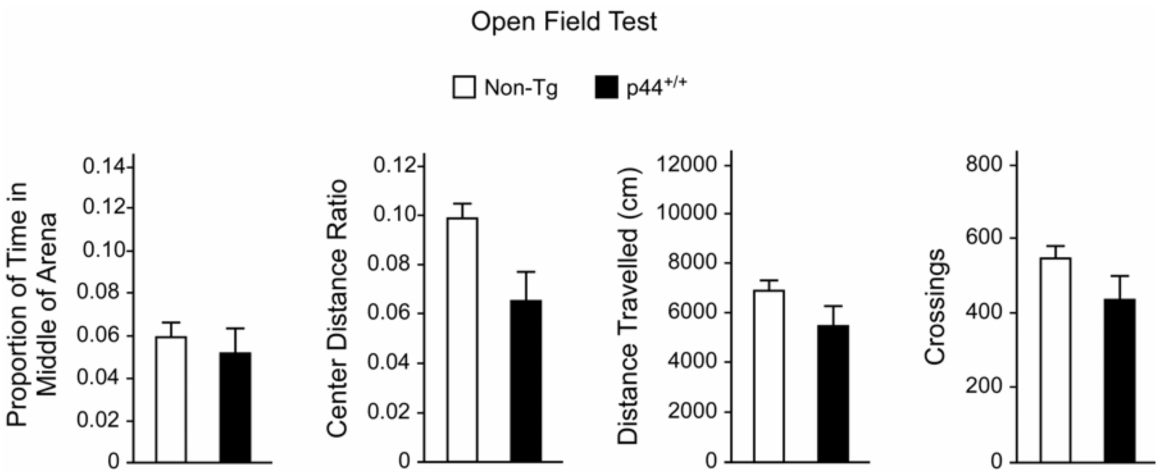

Suppl. Fig. 1. Open Field Test of p44$^{+/+}$ transgenic mice.

Neither anxiety-related responses (proportion of time in the middle of the arena and center distance ratio; *left two panels*) nor measures of motor behavior (total distanced traveled and crossings; *right two panels*) appeared to differentiate 2.5-month-old p44$^{+/+}$ from age-matched Non-Transgenic (Non-Tg) mice. p44$^{+/+}$, n = 9; Non-Tg, n = 14. Values are mean $\pm$ SEM.

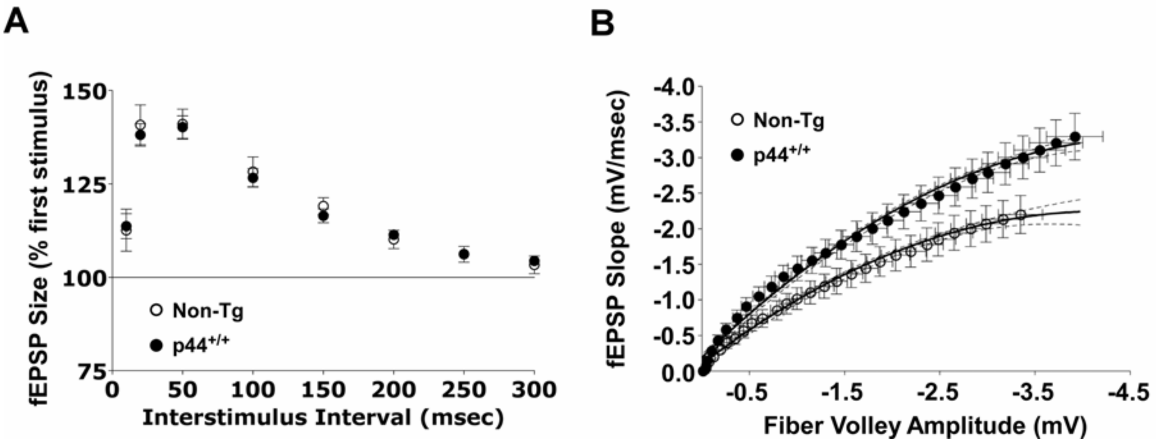

Suppl. Fig. 2. Short-term facilitation and basal synaptic transmission in $p44^{+/+}$ mice.
(**A**) Paired-pulse facilitation in $p44^{+/+}$ and Non-Tg mice across a range of interstimulus intervals (0-300msec). Results are plotted as the ratio of fEPSP slopes (2nd stimulus/1st stimulus X100) as a function of intersimulus interval.

(**B**) Input-output curves in $p44^{+/+}$ and Non-Tg animals. i/o curves were generated by plotting the fEPSP slope as a function of the fiber volley amplitude ($p44^{+/+}$, n = 37(12); Non-Tg, n = 11(5)). No significant difference was observed at 50% magnitude.
Values are mean ± SEM.

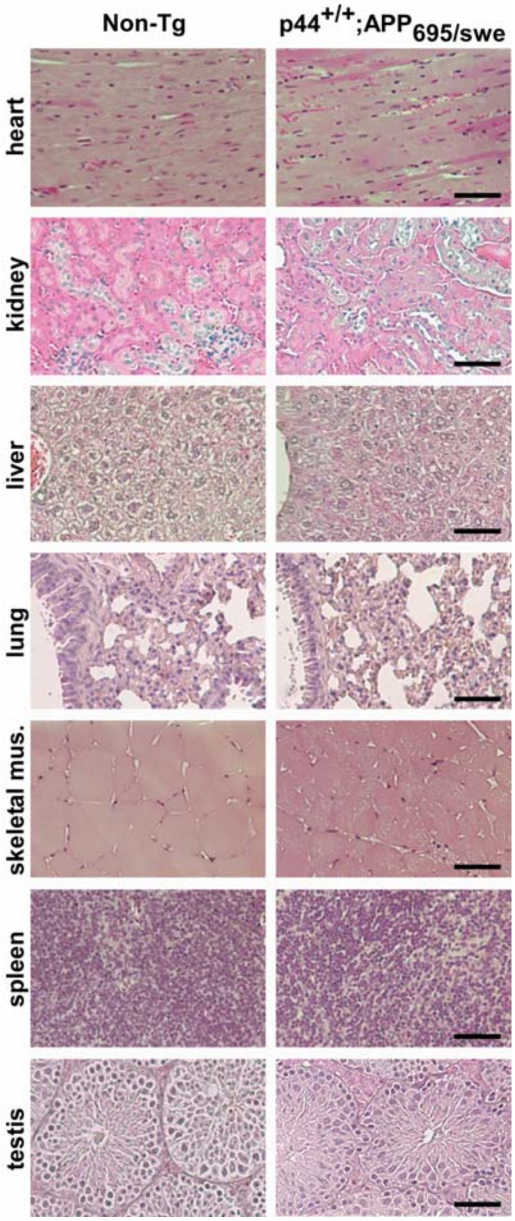

Suppl. Fig. 3. Histology of peripheral organs from Non-Tg and p44$^{+/+}$;APP$_{695/swe}$ double-transgenic mice.

Hematoxylin and eosin stained sections of the indicated peripheral organs/tissues from Non-Tg and p44$^{+/+}$;APP$_{695/swe}$ animals are shown. The double-transgenic mice displayed no obvious pathology. Bar (10X): 60 µm.

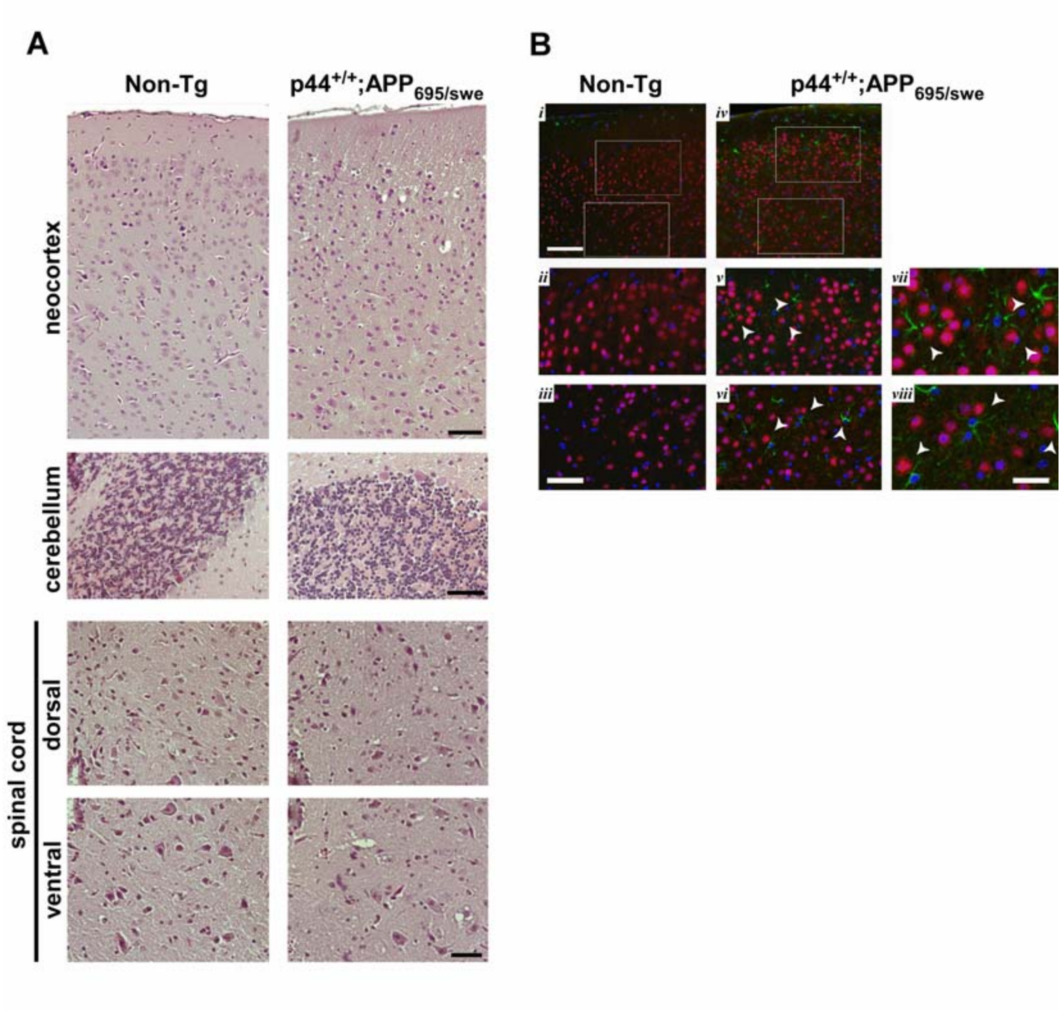

Suppl. Fig. 4. Histology of neocortex, cerebellum, and spinal cord of Non-Tg and $p44^{+/+}$;$APP_{695/swe}$ double-transgenic mice.

(**A**) Hematoxylin and eosin stained sections of the indicated CNS regions of 2.5-month-old Non-Tg and $p44^{+/+}$;$APP_{695/swe}$ mice. The histology of $p44^{+/+}$ and $APP_{695/swe}$ mice is not shown here because it did not reveal any alteration. Sparse eosinophilic neurons with dense cytoplasm could only be observed in the external layers of the neocortex of the double-transgenics. Scale bar: 160 μm (neocortex) and 60 μm (cerebellum and spinal cord).

(**B**) Immunostaining with anti-GFAP (astrocytic marker; green) and anti-NeuN (neuronal marker; red) antibodies. Nuclei were counterstained with DAPI (blue). Microphotographs

show a modest astrogliosis in $p44^{+/+}$;$APP_{695/swe}$ transgenic mice that was absent in Non-Tg animals (compare ***i*** and ***iv***). Microphotographs (***ii***-***iii***) and (***v***-***vi***) show higher magnification of the indicated areas of Non-Tg and $p44^{+/+}$;$APP_{695/swe}$ animals, respectively. Arrowheads indicate the characteristic morphology of reactive astrocytes. (***vii***-***viii***) show higher magnification of reactive astrocytes indicated in (***v***-***vi***), respectively. Bar in (***i***) = 160 µm for (***i*** and ***iv***). Bar in (***iii***) = 80 µm for (***ii***-***iii***) and (***v***-***vi***). Bar in (***viii***) = 40 µm for (***vii***-***viii***).

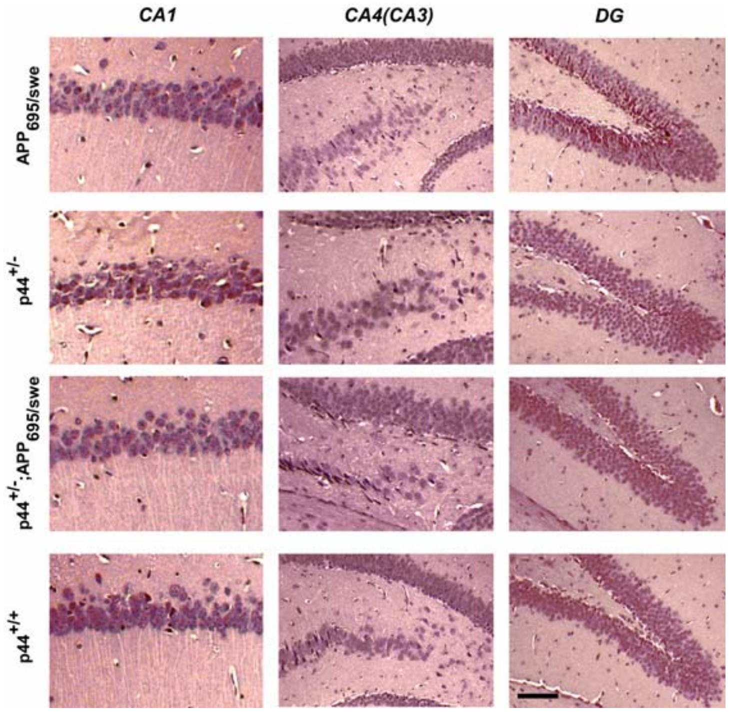

Suppl. Fig. 5. Histology of the hippocampus and dentate gyrus of $APP_{695/swe}$, $p44^{+/-}$, $p44^{+/-}$;$APP_{695/swe}$, and $p44^{+/+}$ animals.

Microphotographs showing different regions of the hippocampus and dentate gyrus. Bar (10X): 100 µm. *DG*: Dentate Gyrus; *CA4(CA3)* is used to indicate the part of *CA3* that inserts into the dentate gyrus. Animals were 2.5-month-old when analyzed.

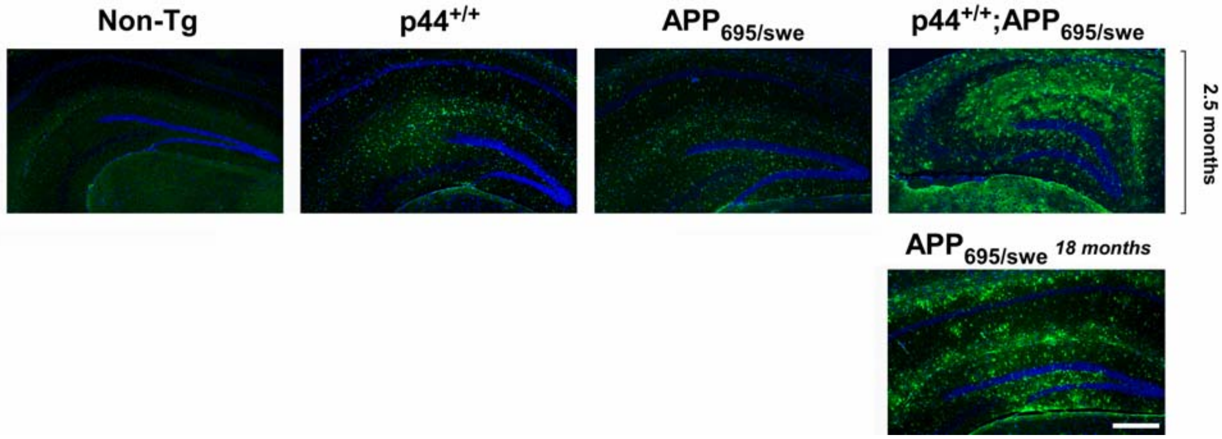

Suppl. Fig. 6. Widespread astrogliosis in the hippocampus and dentate gyrus of $p44^{+/+}$;$APP_{695/swe}$ transgenic mice.

Brain sections of 2.5-month-old Non-Tg, $p44^{+/+}$, $APP_{695/swe}$, and $p44^{+/+}$;$APP_{695/swe}$ mice toghether with 18-month-old $APP_{695/swe}$ mice were immunostained with anti-GFAP (astrocytic marker; green) antibodies. Nuclei were counterstained with DAPI (blue). Scale bar: 400 μm. GFAP immunoreactivity in Non-Tg and $p44^{+/+}$;$APP_{695/swe}$ mice is already shown in Fig. 5A; it is shown here again to allow comparison with age-matched littermates and 18-month-old $APP_{695/swe}$ single-transgenics.

Note the different distribution of GFAP immunoreactivity in $p44^{+/+}$;$APP_{695/swe}$ double-transgenic animals as compared to old $APP_{695/swe}$ single-transgenics. In old $APP_{695/swe}$ mice GFAP immunoreactivity appeared mostly limited to small areas surrounding amyloid plaques (see Fig. 8C).

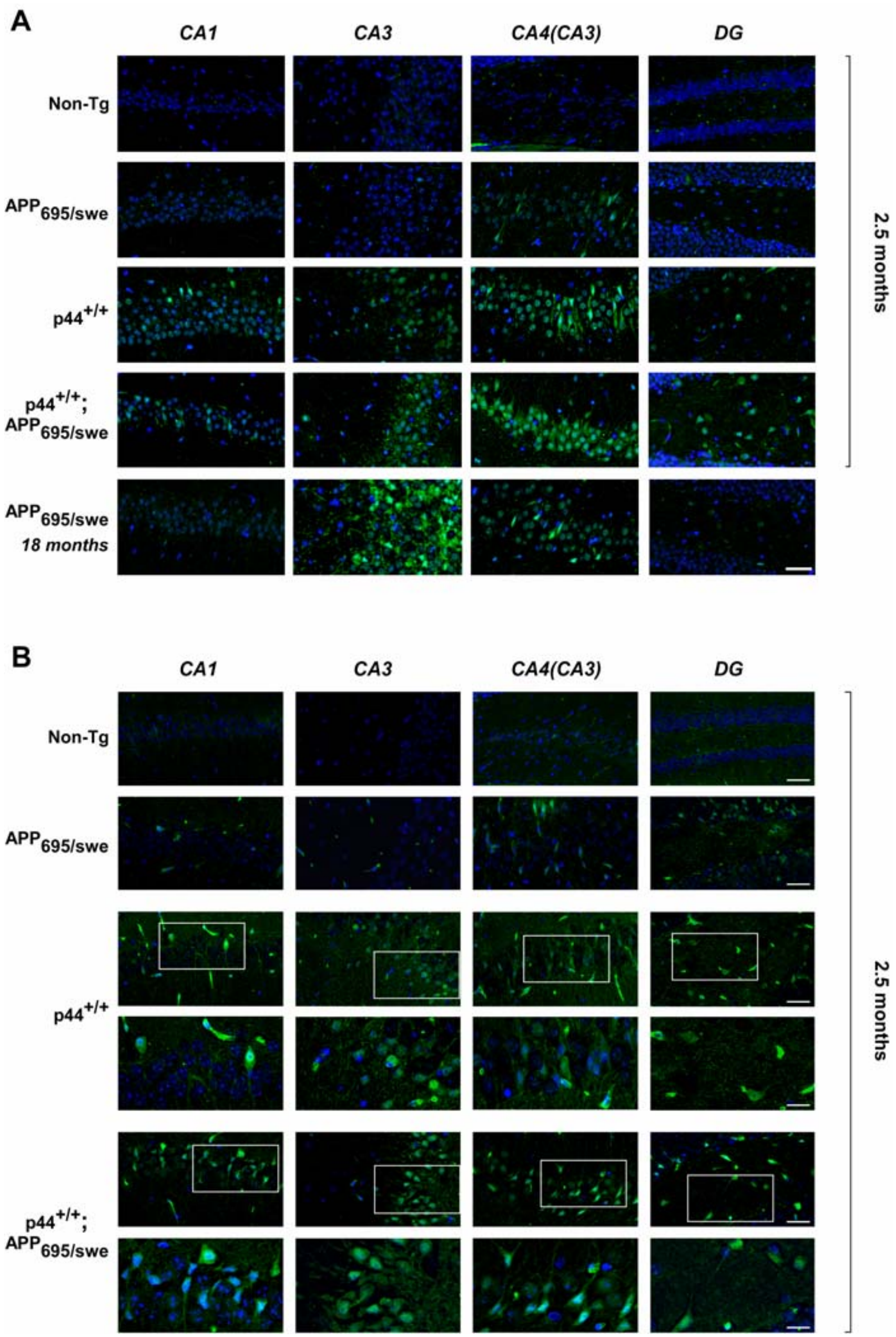

Suppl. Fig. 7. Abnormal phosphorylation of the microtubule-associated protein tau in the hippocampus and dentate gyrus of $p44^{+/+}$;$APP_{695/swe}$ transgenic mice.

(**A**) Brain sections of 2.5-month-old Non-Tg, $APP_{695/swe}$, $p44^{+/+}$, and $p44^{+/+}$;$APP_{695/swe}$ mice toghether with 18-month-old $APP_{695/swe}$ mice were immunostained with anti-phopshorylated tau protein (AD-epitope AT8; against p-Ser202 and p-Thr205). Nuclei

were counterstained with DAPI (blue). Scale bar: 40 µm. High-magnification of Non-Tg, $p44^{+/+}$, and $p44^{+/+}$;$APP_{695/swe}$ are shown in Fig. 3A and 5B, respectively.

(**B**) The same brain areas were immunostained with a different anti-phopshorylated tau protein (against p-Ser356). Nuclei were counterstained with DAPI (blue). Scale bar: 40 µm. Lower panels in $p44^{+/+}$ and $p44^{+/+}$;$APP_{695/swe}$ animals show higher magnification of the indicated areas. Scale bar: 20 µm.

Note that the pattern of tau phosphorylation in $p44^{+/+}$ single-transgenics and $p44^{+/+}$;$APP_{695/swe}$ double-transgenics was remarkably similar (A and B). In contrast, age-matched $APP_{695/swe}$ did not display significant AT8 immunoreactivity (A). In fact, tau hyperphosphorylation could only be observed in old $APP_{695/swe}$, although the pattern was different from $p44^{+/+}$ and $p44^{+/+}$;$APP_{695/swe}$ littermates (A).

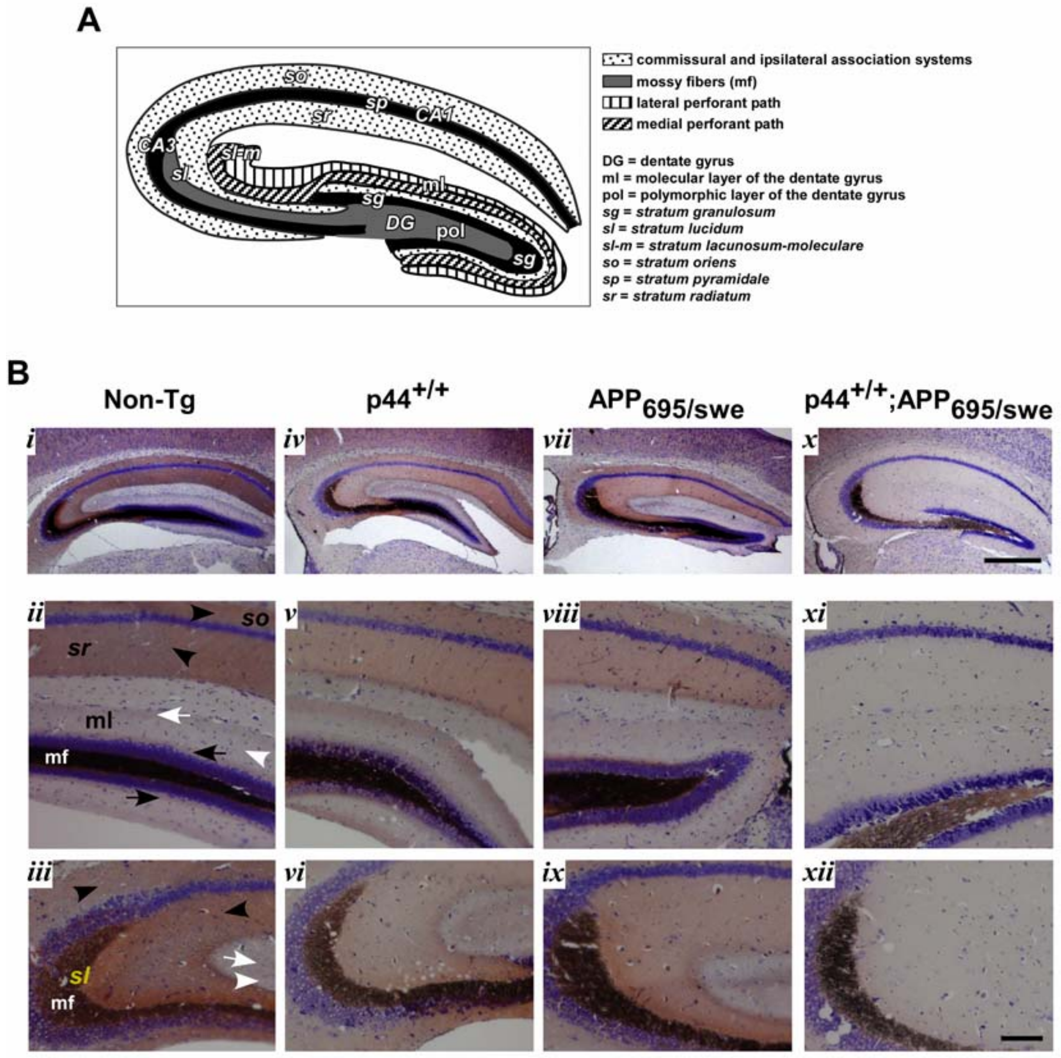

Suppl. Fig. 8. Loss of Timm's staining in p44$^{+/+}$;APP$_{695/swe}$ double-transgenic mice.
(**A**) Schematic representation to illustrate the laminar appearance of the synaptic fields revealed by Timm's staining.
(**B**) Timm's sulfide silver staining of non-transgenic (Non-Tg; ***i*- *iii***), homozygous p44 (p44$^{+/+}$; ***iv*-*vi***), APP$_{695/swe}$ (***vii*-*ix***), and double-transgenic p44$^{+/+}$;APP$_{695/swe}$ mice (***x*-*xii***). ***i*-*iii*** (Non-Tg) and ***x*-*xii*** (p44$^{+/+}$;APP$_{695/swe}$) images are also shown in Fig. 6A. ***v*-*vi*** and ***viii*-*ix*** are higher magnification of ***iv*** and ***vii***, respectively. The mossy fibers (mf), the commissural and ipsilateral association systems (black arrows and arrow-heads) and the perforant pathway (white arrows and arrow-heads) are clearly detectable in p44$^{+/+}$ and

$APP_{695/swe}$ transgenic mice. Abbreviations: mf: mossy fibers; ml: dentate molecular layer; *sl*: CA3 *stratum lucidum*; *so*: *stratum oriens*; *sr*: *stratum radiatum*. Scale bars: 500 µm (upper panel) and 100 µm (lower panels). Timm's staining was performed in 1.5-month-old animals to detect early changes, prior to widespread neurodegeneration.

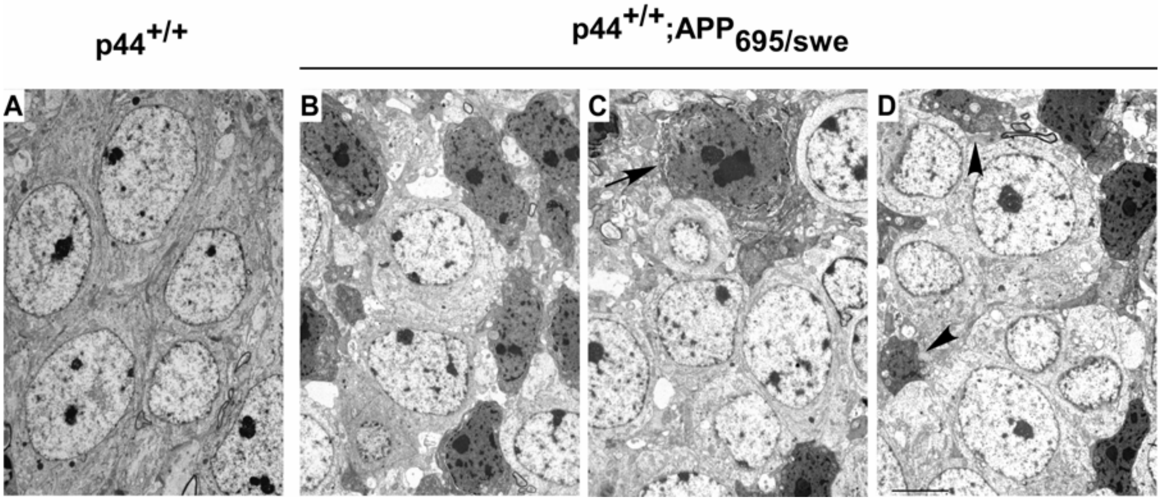

Suppl. Fig. 9. Evidence of paraptosis- and autophagy-like cell death in the hippocampus of $p44^{+/+}$;$APP_{695/swe}$ double transgenic mice.

Electron micrographs of hippocampal sections from 2.5-months old $p44^{+/+}$ and $p44^{+/+}$;$APP_{695/swe}$ double transgenic mice. In $p44^{+/+}$ mice **(A)** the neuropil appears compacted. Neurons have a smooth cell surface and nucleolemma; organelles, including mitochondria, have normal appearance. In contrast, $p44^{+/+}$;$APP_{695/swe}$ mice **(B-D)** display vacuolated interstitial spaces and distended processes surrounding degenerating neurons. Some neurons have a lighter electron dense cytoplasm (compare to $p44^{+/+}$ in A) and display features similar to those observed in cells suffering from paraptosis-like degeneration (see Fig. 9A). In contrast, other neurons display the typical features of dark cell degeneration with highly electron dense cytoplasm and nucleus (see Fig. 9B). Arrow in **C** indicates a neuron in the final stages of degeneration with severe disruption of the nucleolemma. Cells displaying the typical features of microglial cells (small nucleus with crenated nucleolemma and electron dense cytoplasm containing vacuoles; arrowheads in **D**) were evident in the double-transgenics. Scale bar: 5µm.

REFERENCES FOR SUPPORTING INFORMATION

Costantini C, Scrable H, Puglielli L (2006) An aging pathway controls the TrkA to p75(NTR) receptor switch and amyloid beta-peptide generation. *EMBO J*. **25**, 1997-2006.

Costantini C, Weindruch R, Della Valle G, Puglielli L (2005) A TrkA-to-p75NTR molecular switch activates amyloid beta-peptide generation during aging. *Biochem J*. **391**, 59-67.

Hsiao K, Chapman P, Nilsen S, Eckman C, Harigaya Y, Younkin S, Yang F, Cole G (1996) Correlative memory deficits, Abeta elevation, and amyloid plaques in transgenic mice. *Science* **274**, 99-102.

Irizarry MC, McNamara M, Fedorchak K, Hsiao K, Hyman BT (1997) APPSw transgenic mice develop age-related A beta deposits and neuropil abnormalities, but no neuronal loss in CA1. *J Neuropathol Exp Neurol*. **56**, 965-973.

Lesne S, Koh MT, Kotilinek L, Kayed R, Glabe CG, Yang A, Gallagher M, Ashe KH (2006.) A specific amyloid-beta protein assembly in the brain impairs memory. *Nature* **440**, 352-357.

Liu JP, Baker J, Perkins AS, Robertson EJ, Efstratiadis A (1993) Mice carrying null mutations of the genes encoding insulin-like growth factor I (Igf-1) and type 1 IGF receptor (Igf1r). *Cell* **75**, 59-72.

Maier B, Gluba W, Bernier B, Turner T, Mohammad K, Guise T, Sutherland A, Thorner M, Scrable H (2004) Modulation of mammalian life span by the short isoform of p53. *Genes Dev*. **18**, 306-319.
